# The international diffusion of food innovations and the nutrition transition: retrospective longitudinal evidence from country-level data, 1970–2010

**DOI:** 10.1136/bmjgh-2023-012062

**Published:** 2023-11-15

**Authors:** Anne-Célia Disdier, Fabrice Etilé, Lorenzo Rotunno

**Affiliations:** 1 Paris School of Economics, Paris, France; 2 UMR 1393, Paris-Jourdan Sciences Economiques, INRAE, Paris, France; 3 Aix-Marseille Univ, CNRS, AMSE, Marseille, France

**Keywords:** Nutrition, Prevention strategies, Public Health, Cohort study

## Abstract

**Introduction:**

There is a lack of quantitative evidence on the role of food innovations—new food ingredients and processing techniques—in the nutrition transition.

**Objective:**

Document the distribution of food innovations across 67 high-income (HIC) and middle-income (MIC) countries between 1970 and 2010, and its association with the nutritional composition of food supply.

**Methods:**

We used all available data on food patents, as compiled by the European Patent Office, to measure food innovations. We considered innovations directly received by countries from inventors seeking protection in their territories, and those embedded in processed food imports. Food and Agricultural Organization data were used to estimate the associations between international diffusion of food innovations and trends in total food supply and its macronutrient composition, after adjusting for confounding trends in demand-side factors. We identified the role of trade by simulating the changes in average diet due to innovations embedded in food imports.

**Results:**

Trends in food innovations were positively and significantly associated with changes in daily per capita calorie supply available for human consumption in MIC between 1990 and 2010 (elasticity of 0.027, 95% CI 0.019 to 0.036). Food innovations were positively correlated with the share of animal and free fats in total food supply (elasticities of 0.044, 95% CI 0.030 to 0.058 for MIC between 1970 and 1989 and 0.023, 95% CI 0.003 to 0.043 for HIC between 1990 and 2010). Food innovations were associated with substitutions from complex carbohydrates towards sugars in total food supply for MIC after 1990 (elasticities of −0.037, 95% CI −0.045 to −0.029 for complex carbs, 0.082, 95% CI 0.066 to 0.098 for sugars). For these countries, the trade channel capturing access to innovations through imports of processed food played a key role.

**Conclusion:**

Policy-makers should consider the impacts of the international diffusion of food innovations in assessing the costs and benefits of international trade regulations.

**WHAT IS ALREADY KNOWN ON THIS TOPIC**

- While previous research has shown that economic and social globalisation are key drivers of the nutrition transition, it has not systematically explored the role of one key component of globalisation—the international diffusion of food innovations—in variations in the nutritional quality of food supply across countries and over time.

**WHAT THIS STUDY ADDS**

- International diffusion of food innovation is associated to a decrease in the share of complex carbohydrates in total food supply, and an increase in the share of sugars.
- The large positive association with the increasing share of sugar in middle-income countries over the globalisation period 1990–2010 is explained by an extended access to innovations embedded in imports of processed food products.
- These associations are robust to the inclusion of demand-side pulling factors, such as income growth, urbanisation, and changes in women labour-market participation.

**HOW THIS STUDY MIGHT AFFECT RESEARCH, PRACTICE OR POLICY**

- While current trade policies account for food safety within the World Trade Organization (WTO) regulation framework, countries should also be allowed to enforce trade regulatory measures dealing with the nutritional quality of food imports.

## Introduction

Food systems experienced a profound shift in high-income (HIC) and middle-income (MIC) countries over the past forty years. Agri-food production and processing techniques rapidly evolved, leading to significant quantitative and qualitative changes in food supply. These changes can be observed in the macronutrient composition of daily calories available for human consumption, which can be derived from the Food and Agricultural Organization (FAO) data. Total calorie supply significantly increased from 2389 to 2850 kcal per capita par day between 1970 and 2010 (our period of analysis), implying that food security improved at a population level. Yet, calorie composition has been dramatically altered. During that period, calorie supply from free fat (eg, fat from oil, butter and cream) rose by 30% in HIC, 78% in lower MIC and doubled in upper MIC. Calories from sugars increased by 25% in lower MIC and by 23% in upper MIC.^1 2^ These alterations in the calorie composition of food supply characterise a nutrition transition and pose important policy challenges for global health.^3 4^

Over the last decades, multiple path-breaking food innovations have emerged. In the 1970s, the division of the production process into two stages revolutionised the food industry. During the first stage, raw agricultural products are broken down to extract elementary ingredients (eg, oils, fats, flours, animal fats, lactose). In the second stage, the ingredients are recombined—often with additional chemicals such as artificial colourants and stabilisers—to formulate final consumer products. The creation of products no longer occurs at the agricultural level but is pushed down to the second stage, and facilitates innovation and emergence of new varieties.^2 5^ For the purpose of this study, food innovations are measured by patents classified under the food processing and food chemistry sectors.

Additional innovations boosted the growth of food markets. The development of cold chains made long distance trade of non-stabilised products possible. Various technological advancements helped to preserve food products on shelves. These improvements and a sharp fall in transportation costs led to the globalisation of food chains, which now involve different actors specialised in specific steps.^6^ Advances in food preservation and logistics also facilitated the expansion of large and concentrated retailing chains. Supermarkets emerged in Latin America in the mid-1980s, in Southeast Asia in the mid-1990s to late-1990s and in China, Vietnam, India and Russia in the late 1990s and 2000s. They expanded recently in Eastern and Southern Africa.^7 8^

All these changes altered the price and quality of products offered to consumers. Market studies by nutritionists emphasised that calorie-dense and processed foods are deficient in essential nutrients; rich in sugar, fat and salt; and cheaper in terms of cost per calorie than healthy products.^9 10^ Processed foods are major contributors to deviation of household purchases from dietary recommendations, in HIC and in MIC.^11–15^

These shifts in consumption patterns raise the issue of implementing more stringent regulations on food technologies. An extensive literature in public health documented the role of food innovations in the nutrition transition.^3^ The evidence so far is essentially based on country-specific case studies, and does not account for demand-side factors—such as women labour force participation, urbanisation or globalisation-induced changes in food preferences—that may confound the association between innovations and nutritional outcomes.^1 16–19^

Using a sample of 38 HIC and 29 MIC, this study addressed two objectives. The first objective was to characterise food innovations across countries and for two subperiods 1970–1989 and 1990–2010, the latter being marked by an acceleration of globalisation. We considered all innovations used for the transformation of raw agricultural commodities into food products for human consumption. We examined innovations directly received by countries from inventors requesting protection and those indirectly obtained through imports of processed food products. The second objective was to identify the association between trends in innovations and variations in calorie supply and its composition, after adjusting for the confounding effects of changes in demand-side factors. We translated the estimated results, first by simulating the expected changes in the average diet of countries if the number of innovations raised up to the maximum observed in their income-group (the ‘technological frontier’), and second by a counterfactual exercise illustrating the role of innovations embedded in imports of processed food.

## Methods

### Overview

We combined different sources of data to document the diffusion of food technologies across countries and over time, and its associations with changes in country-level nutritional outcomes. Our final dataset included 38 HIC and 29 MIC in the period 1970–2010. The HIC and MIC groups were defined using the 2014 World Bank income classification. The set of countries was restricted to HIC and MIC that had food-related patent applications, and for which we observed patent, nutrition, trade and other data used in the analysis for at least 16 years during the sample period. The full sample selection process, as well as countries present in the sample are detailed in online supplemental appendix p 3, pp 10–11 and p 12. We eliminated observations after 2010 because application data were incomplete after that date in the PATSTAT database acquired in 2018. Indeed, some national patent offices communicate application data with a significant delay, for various reasons including application examination procedure, confidentiality, etc (online supplemental appendix p2).

10.1136/bmjgh-2023-012062.supp1 Supplementary data

### Patient and public involvement

It was not appropriate to involve public in our research.

### Exposure: food innovations

We used patents as a measure of innovations available in each country. We attributed patents to countries by using the designated state(s) in which an (domestic or foreign) inventor asks for protection. We drew our patent data from the Worldwide Patent Statistical Database (Patstat), maintained and diffused by the European Patent Office—EPO (online supplemental appendix pp 1–2 and p 10). This database covers almost all worldwide patents since the 1950s. We first kept only patents with International Patent Classification (IPC) codes related to food processing and food chemistry techniques. This includes IPC codes that directly relate to food products, but also those that refer to the extraction of fat or sugar molecules, ingredients or products (online supplemental appendix p20). We did not select innovations in agriculture, animal husbandry or fishing, as they are not necessarily related to human food consumption. We then restricted the sample to patents for which at least one of the industrial domains of application is the manufacturing of food products or beverages, as defined in the Patstat database. We further counted patents at the level of the ‘patent family’. A family is a set of patents that are related to each other by one or several common priority dates filed but may be filed at various national or international offices.

Since we were only interested in significant innovations, we kept patent families with applications filed in at least two of the following key patent offices: EPO and its main member states (France, Germany, Italy, Netherlands, Spain, Switzerland and UK), US Patent Office, Australia, Brazil, Canada, China, Japan, South Korea, Mexico, South Africa and Soviet Union/Russia. These offices cover the origin countries of the top multinational food companies, which concentrate most patented food technologies.^20^ Last, we used information on the earliest filing date at national patent offices to know when the innovation arrived in a country. Following usual research practices, we did not use any information on whether the patent was effectively granted or not.^21^ This information is indeed very patchy, as national offices do not always provide it to the EPO, and the granting lag as well as the evaluation standards are highly heterogeneous across offices and over time.^22 23^ We defined the annual stock of food innovations present in each country as the sum of past cumulated flows of patents (online supplemental appendix p7). We focused on patents registered since 1950.

Countries can obtain innovations through two main channels: directly, from domestic and foreign inventors asking for protection in their territories and indirectly through food imports. We measured exposure to patents filed in foreign countries through imports of processed foods, as these are likely to embed food innovations. The stock of imported innovations in a country was computed as the weighted sum of patent applications filed in the country’s trading partners. The weights were equal to the partner share in the country’s total imports of processed foods (online supplemental appendix p 7). Import data were extracted from the United Nations Comtrade database (online supplemental appendix p 4).

### Nutritional outcomes

Countries were positioned in their nutrition transition using indicators on the quantity and the quality of their food supply between 1970 and 2010. First, relying on food balance sheets from the FAO (online supplemental appendix pp 3–4), we considered the average total calorie supply per capita and per day available for human consumption. Second, we computed the shares in average per capita and per day calorie supply of main nutrients (carbohydrates, fats and proteins), and their subcomponents (complex carbohydrates vs sugars, vegetable fats vs free or animal fats, vegetable proteins vs animal proteins).

### Socioeconomic indicators and covariates

We conditioned our analysis on a large set of demand-side control variables, including country gross domestic product (GDP) per capita, women labour force participation and urbanisation rate. These control variables were obtained from the World Bank World Development Indicators database (online supplemental appendix pp 4–5). We also considered indicators of social and economic globalisation. We used the subindices of the KOF Index of globalisation developed by Dreher^24^, and updated by Gygli *et al*.^24 25^ The economic globalisation index captures changes in economic flows (trade, foreign direct investments, portfolio investments, income payments to foreign nationals) and trade openness (hidden import barriers, mean tariff rate, taxes on international trade, capital account trade openness). Social globalisation is constructed from data on personal contacts (telephone traffic, transfers, international tourism, share of foreign population and international letters), information flows (internet and telephone users, trade in newspapers), and cultural proximity (number of McDonald’s restaurants, Ikea stores, trade in books) (online supplemental appendix p 5). Finally, relying on FAO food balance sheets, we included the share of food imports in total calorie supply, as this variable is likely to have a direct impact of nutritional outcomes, beyond its indirect impact captured through the food innovation measure.

### Statistical analysis

We first estimated the contribution of food technologies’ diffusion to the nutrition transition, by regressing the total per capita and per day food supply and the shares of macronutrients on the stock of patents per country year. Logarithmic or logit transformations were applied to outcome variables. These estimates provide a basis for consistently analysing trends in nutrition transition related to food innovations and for highlighting correlations.

Patenting activity depends on several factors that may affect the identification of a causal effect. Companies may decide to file a patent application for developing processes or products that will meet or increase local consumer demand. Thus, demand-side factors are likely to pull food innovations and may confound the estimated association. Consequently, we included a large set of demand-side control variables (GDP per capita, female labour force participation, urbanisation rate) in our analysis. We also adjusted for trends in social and economic globalisation, and for changes in the share of food imports because our measure of innovations is, by construction, correlated with this determinant of food supply. Some country-year observations had a zero patent count. These zeros can be true zeros or misreporting, especially if the country was not a member of the Patent Cooperation Treaty (PCT) or the Paris Convention. To control for these zeros, we added two additional covariates to our regressions: a dummy controlling whether the country was a member of the PCT in a given year (0 otherwise), and a dummy for the country’s membership to the Paris Convention. Finally, we added country fixed effects and year fixed effects. In the regressions on MIC, year fixed effects were further interacted with income group (upper MIC and lower MIC) fixed effects. Our estimates thus reflected the association between within-country changes in the total stock of food innovations and within-country changes in nutritional outcomes conditional on within-country variations in the covariates and income-group specific year shocks. Details on model specification and statistical analyses are presented in online supplemental appendix Section B (pp 7–9).

We then performed two counterfactual exercises. First, we simulated the impact of an increase in the number of innovations up to the technological frontier on nutrition trends by group of countries (HIC vs MIC) and subperiods (1970–1989 vs 1990–2010). The reference used for these simulations was a rise in the number of innovations up to the maximum number observed in our sample within the same income group and same time period. Additionally, we investigated the contribution of international trade to changes in nutrition trends. We computed the relative variation in average nutritional outcomes when adding innovations indirectly obtained through import flows of processed food products to the innovations received directly from investors seeking protection in the domestic territory.

A series of sensitivity analyses were performed (online supplemental appendix pp 25–28). We first tested the consistency of our results across different model specifications, including several sets of controls (base controls, membership to PCT and Paris Convention, and additional controls) and fixed effect combinations. We then tested the robustness of our results on a balanced sample of countries (25 HIC and 18 MIC). Finally, we repeated the estimations focusing on innovations directly received from domestic and foreign inventors seeking for protection on the domestic market and excluding innovations indirectly obtained through imports of processed food products. The sensitivity of the simulation of an increase in innovations up to the technological frontier was also investigated by computing the counterfactual variations without accounting for PCT and Paris Convention memberships (online supplemental appendix p 17).

Statistical analyses were done using Stata, V.17. SEs were clustered by country.

### Role of the funding source

The funders of the study had no role in the study design, data collection, data analysis, data interpretation, or writing of the report.

## Results

Our final estimation sample included 38 HIC and 29 MIC between 1970 and 2010. We considered two distinct subperiods (1970–1989 and 1990–2010), years 1990s and 2000s being marked by hyperglobalisation. An overview of food supply and its components, food innovations and country-level characteristics is reported in table 1, and additional descriptive statistics are presented in online supplemental appendix pp 13–16.

**Table 1:** Sample characteristics

Countries and time periods	HIC, 1970–1989		HIC, 1990–2010		MIC, 1970–1989		MIC, 1990–2010	
	Mean	SD	Mean	SD	Mean	SD	Mean	SD
Food supply (kcal/capita/day)	3130.4	241.6	3246.2	271.5	2436.0	386.4	2728.5	417.6
Carbohydrates (% food supply)	54.5	7.1	53.5	5.6	70.4	4.0	66.9	5.1
Complex carb. (% food supply)	29.9	9.8	29.6	6.7	48.8	9.2	46.9	8.7
Sugars (% food supply)	24.6	3.8	23.9	3.1	21.7	7.6	19.9	5.6
Fats (% food supply)	33.4	6.8	34.2	5.3	19.6	3.8	22.6	4.7
Vegetable fats (% food supply)	2.5	0.8	2.8	0.8	4.1	1.6	4.1	1.6
Free or animal fats (% food supply)	30.9	7.2	31.4	5.4	15.5	4.5	18.5	5.7
Proteins (% food supply)	12.1	0.9	12.2	0.9	9.9	1.0	10.6	0.9
Vegetable proteins (% food supply)	5.1	1.1	5.1	0.7	6.9	1.5	6.8	1.3
Animal proteins (% food supply)	7.1	1.6	7.1	1.2	3.0	1.2	3.8	1.3
Total patents (weighted count)	1566.3	1422.0	3729.8	3515.8	387.7	400.2	1341.6	1139.8
Patents directly received (count)	372.4	388.4	1206.3	1175.4	340.5	401.6	971.2	901.9
Patents imported *via* trade (weighted count)	1455.0	1632.9	3127.3	4023.4	51.9	135.0	422.7	911.8
Countries without patents directly received (%)	10.2	1.3	2.6	0.6	63.9	2.4	8.7	1.2
GDP per capita (US$1000, 2005)	23.2	0.5	29.5	0.6	3.1	0.1	3.9	0.1
Female labour force participation (%)	40.3	0.5	50.3	0.3	26.9	0.7	42.2	0.6
Urbanisation rate (%)	74.2	0.5	76.0	0.4	46.0	0.7	55.7	0.6
Social globalisation (0–100)	63.7	0.6	71.7	0.4	29.8	0.5	42.3	0.6
Economic globalisation (0–100)	44.2	0.8	60.4	0.6	35.1	0.7	49.6	0.7
Food imports (% food supply)	21.7	0.8	23.8	0.7	20.3	0.9	24.0	0.8
PCT (% members)	32.4	2.0	86.4	1.2	3.1	0.9	56.6	2.1
Paris convention (% members)	90.4	1.3	97.2	0.6	51.7	2.5	90.6	1.2

Note: ‘patents directly received’ are patent applications directly received by countries from domestic and foreign inventors asking for protection in the domestic territory. ‘Patents imported via trade’ are patent applications indirectly obtained by countries through imports of processed food products. ‘Total patents’ are computed as the weighted sum of ‘patents directly received’ and ‘patents imported via trade’. ‘Countries without patents directly received’ is the share of countries without ‘patents directly received’.

GDP, gross domestic product; HIC, high-income country; MIC, middle-income country; PCT, patent cooperation treaty.

While food supply increased in quantity in both groups of countries between 1970–1989 and 1990–2010 (+3.7% for HIC and +12.0% for MIC), the composition of the diet changed, with a decrease in the share of carbohydrates (−1.8% for HIC and −5.0% for MIC) compensated by a rise in the share of fats (+2.4% for HIC and +15.3% for MIC) and proteins (+0.8% for HIC and +7.1% for MIC). Moving to subcomponents, we have also shown a significant increase in the share of free and animal fats (+1.6% for HIC and +19.4% for MIC), as well as in the share of animal proteins (+26.7% for MIC).

These changes were concomitant with a rise in food innovations. The number of total patents obtained by countries increased on average by 138.1% for HIC and 246.0% for MIC. Increasing trends were observed for directly and indirectly received patents. We also observed a strong rise in patents related to ultra-processed food (UPF) products over time. At the end of the period, this share reached more than 40% both in HIC and MIC (online supplemental appendix p 16).

These two subperiods 1970–1989 and 1990–2010 were also characterised by an increase in economic development (GDP per capita: +27.2% for HIC and +25.8% for MIC), a more active participation of women to the labour force (+24.8% in HIC and +56.9% in MIC), more urbanisation (+2.4% in HIC and +21.1% in MIC) and a strong rise in the world integration of countries through social globalisation (+12.6% in HIC and +41.9% in MIC) and economic globalisation (+36.7% in HIC and +41.3% in MIC).

The diffusion of food innovations across countries and years could be studied using a dendrogram analysis (online supplemental appendix p 13–15). It helps grouping countries with similar trends into common clusters and visualising distances between each cluster. Two main patterns emerge. First, a few HIC countries received directly a large number of innovations. Second, other HIC and biggest MIC tended to compensate their deficit in direct innovations through international trade and imports of innovations encapsulated into processed food products.

**Figure 1:**
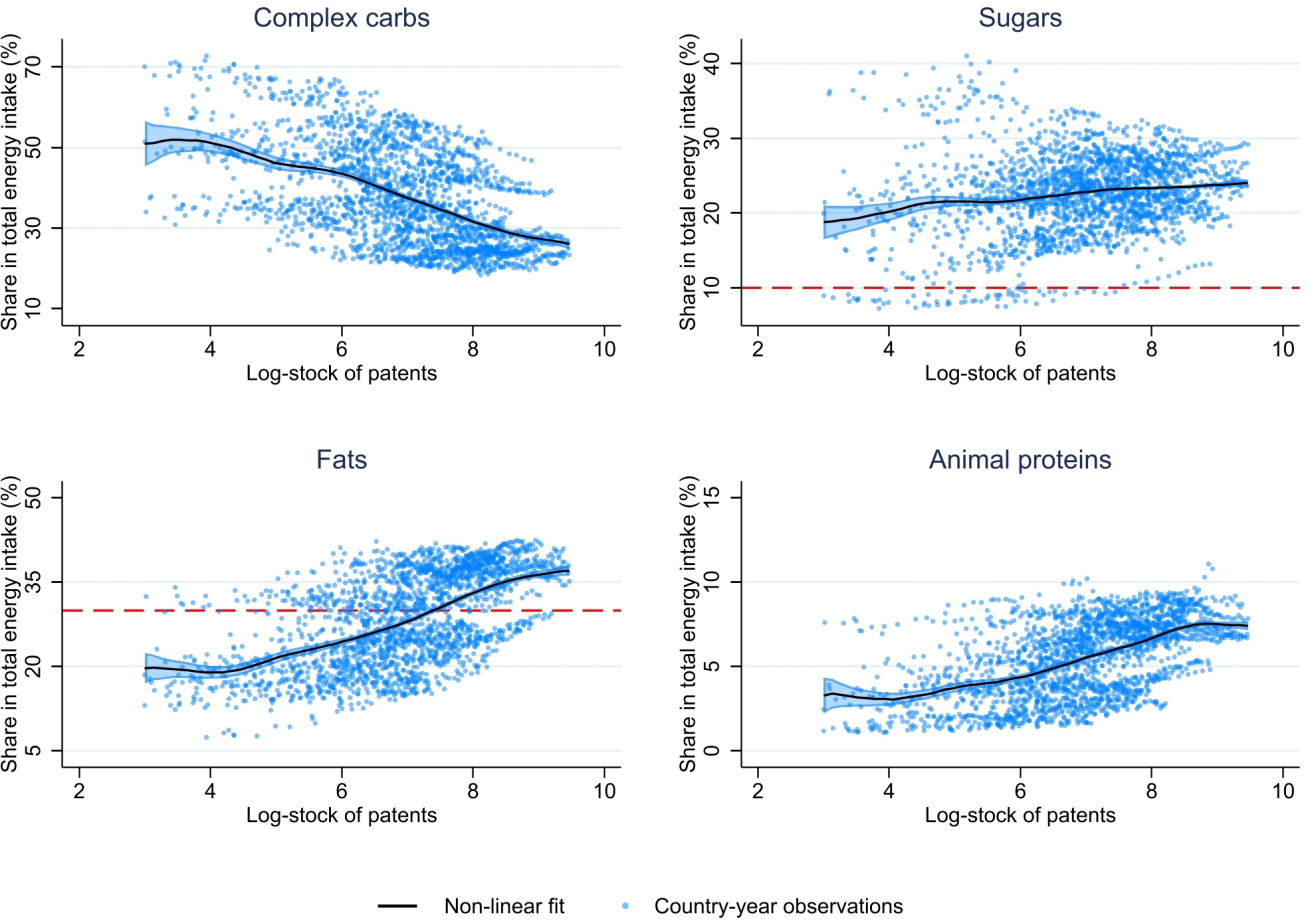
Correlation between innovations and main food supply components. Note: Each dot represents a country-year observation included in our sample. The dotted lines report the FAO reference values for nutrient intake. These values should protect almost all individuals against potential damage to health from their diet and provide a basis for full functional capacity. Total fats should not exceed 30% of energy, and sugars should not provide more than 10% of energy. FAO, Food and Agricultural Organization.

Besides, a positive and significant correlation was observed between the stock of innovations (in logs) and the share of the fats and animal proteins and to a lesser extent for sugars in total calorie supply per capita and per day (in %). By contrast, a negative correlation was shown for complex carbohydrates (figure 1).

Two models were then estimated, one for each group of countries (HIC and MIC) and adjusted for base controls (GDP per capita in logs, GDP per capita squared in logs, share food imports in logs), additional controls (women labour force participation, women labour force participation squared, urbanisation rate, social globalisation, economic globalisation), memberships to PCT and Paris convention and fixed effects (See equation 1 in online supplemental appendix p 8). Elasticities for each subperiod (1970–1989 and 1990–2010) were derived from estimated coefficients (online supplemental appendix p 8 and pp 23–24). Elasticities show the relative increase in the outcome variable (total calorie supply, share of macronutrients) that is associated to a 1% increase in the annual stock of food innovations (table 2). The results highlighted that the association between food innovations and food supply and its main components was heterogeneous across country income groups and subperiods.

**Table 2:** Associations (elasticities) between food innovations received by HIC and MIC and food supply and its main components

Countries	HIC		MIC	
Time period	1970–1989	1990–2010	1970–1989	1990–2010
Ln food supply	−0.004 (0.003)	−0.001 (0.004)	0.006 (0.004)	0.027*** (0.004)
Ln share carbohydrates	0.002 (0.003)	−0.016*** (0.004)	−0.004* (0.002)	0.003 (0.003)
Ln share complex carbohydrates	−0.004 (0.005)	−0.018*** (0.006)	−0.008** (0.004)	−0.037*** (0.004)
Ln share sugars	0.013*** (0.005)	0.001 (0.006)	0.009 (0.007)	0.082*** (0.008)
Ln share fats	−0.003 (0.005)	0.033*** (0.006)	0.016** (0.008)	−0.005 (0.008)
Ln share vegetable fats	0.005 (0.008)	−0.025** (0.010)	0.005 (0.008)	0.037*** (0.009)
Ln share free and animal fats	−0.004 (0.006)	0.044*** (0.007)	0.023** (0.010)	−0.005 (0.011)
Ln share proteins	0.002 (0.003)	−0.011*** (0.004)	−0.004 (0.003)	−0.002 (0.003)
Ln share vegetable proteins	0.006 (0.005)	−0.009 (0.006)	−0.002 (0.004)	−0.011*** (0.004)
Ln share animal proteins	0.001 (0.007)	−0.000 (0.008)	−0.000 (0.008)	0.016* (0.009)
Observations	1243		945	
Countries	38		29	

Note: Estimations include base controls (log(GDP per capita), log(GDP per capita) squared, log(share food imports)), additional controls (women labour force participation, women labour force participation squared, urbanisation rate, social globalisation, economic globalisation), controls (dummies) for Patent Cooperation Treaty and Paris convention memberships. Sets of fixed effects are: country and year fixed effects for estimations on HIC and country and year×income group for estimations on MIC. These results are based on the estimation of equation 1 online supplemental appendix p 8 for each group of countries (HIC and MIC). Elasticities are computed using formula provided in online supplemental appendix p 8.

*p<0.1, **p<0.05, ***p<0.01. Standard errors in parentheses.

GDP, gross domestic product; HIC, high-income country; MIC, middle-income country.

For HIC, a positive association between food innovations and the share of sugars in total calorie supply was observed between 1970 and 1989 and between food innovations and the share of free and animal fats between 1990 and 2010. A doubling in the number of food innovations was linked to an 1.3% increase in the share of sugars for the period 1970–1989 and to a 4.4% increase in the share of free and animal fats for the period 1990–2010. By contrast, a negative correlation was found between food innovations and the share of complex carbohydrates, Vegetable fats and proteins between 1990 and 2010.

For MIC, a positive and significant association was observed between innovations and total food supply between 1990 and 2010: A doubling in the number of food innovations was related to a 2.7% increase in total calorie supply. A positive correlation was also found between innovations and the share of animal and free fats during the first subperiod 1970–1989 and the shares of sugars and vegetable fats during the second subperiod 1990–2010. A doubling in the number of innovations between 1990 and 2010 was linked with an increase equal to 8.2% (respectively, 3.7%) of the share of sugars (respectively, vegetable fats) in total calorie supply. On the other hand, a negative association was found with the share of complex carbohydrates for the two subperiods as well and with the share of vegetable proteins for the second subperiod.

**Figure 2:**
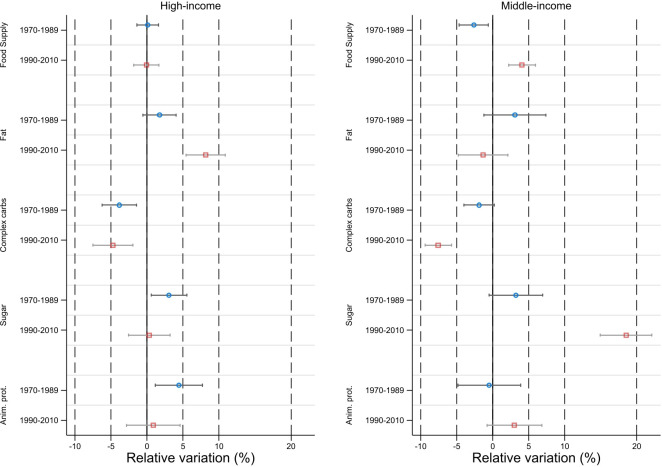
Simulated changes in nutritional outcomes if innovation stock increases up to the technological frontier. Note: Relative variation in average nutritional outcomes for total food supply and selected macronutrients when moving from the observed mean to the observed maximum of total innovations in the same income group, same subperiod.

Then, we simulated the effect of an increase in the number of the food innovations up to the technological frontier, defined as the maximum number of innovations observed in each country within the same income group and same subperiod. We highlighted three main results (figure 2). First, the increase in the number of innovations up to the max appeared to be strongly and positively correlated with the average total calorie supply per capita and per day available for human consumption in MIC and for the second subperiod 1990–2010. By comparison, the correlation with the calorie supply was not significant in HIC. Second, it also induced changes in the quality of the diet, with a significant decrease in the share of complex carbohydrates in the total calorie supply and by contrast a strong rise in the share of sugars. Results on carbohydrates were observed for both HIC and MIC, but the innovation-related shifts were more pronounced for the latter over 1990–2010. Third, results on fats and animal proteins were less salient. In particular, intensifying the diffusion of food innovations everything else equal was not associated to a significant increase in the share of animal proteins for MIC.

**Figure 3:**
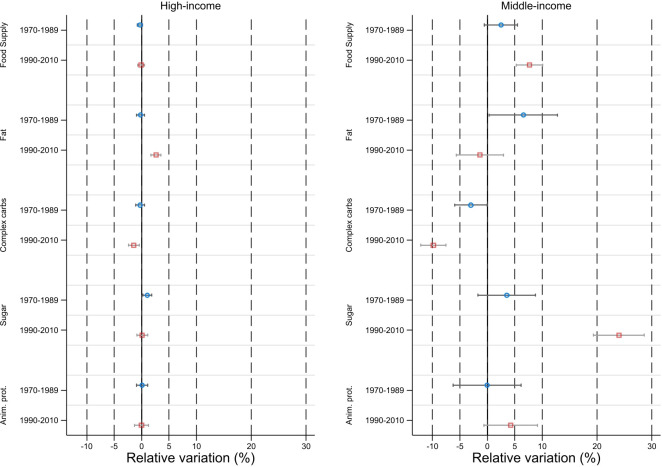
Contribution of international trade to changes in nutritional outcomes. Note: Relative variation in average nutritional outcomes for total food supply and selected macronutrients when adding innovations indirectly obtained through imports of processed food products to innovations directly received from investors seeking protection in the domestic market.

Finally, our second counterfactual exercise showed the decisive role played by the trade channel for MIC (figure 3). Changes in nutritional outcomes observed in these countries were significantly driven by the food innovations indirectly obtained through imports of processed food products from foreign countries and based on innovative production processes. Nutrition outcomes in MIC were impacted not only in quantitative terms (food supply available for consumption) but also in qualitative terms. In particular, international trade induced a very strong increase in the share of sugars in total food supply and a decrease in the share of complex carbohydrates. By contrast, the contribution of international trade was, on average, almost not significant for HIC. Given the size of these markets, foreign and domestic investors tended to directly seek protection for their innovations in these markets and food outcomes in HIC were directly impacted by these innovations rather than by the innovations embodied into imported processed food products.

### Sensitivity analysis

Our models and counterfactual exercises were robust to sensitivity analysis checks (online supplemental appendix pp 17–19 and pp 25–28). Our results did not change under different specifications (exclusion of additional controls, absence of controls for PCT and Paris Convention memberships). Results were also robust to the use of a balanced sample of countries, which allowed us to follow exactly the same set of countries (25 HIC and 18 MIC) over the whole period of analysis. The only significant difference was observed when fixed effects were removed from the estimations. In such case, correlations became insignificant. However, these results were likely to be biased as time-invariant country characteristics and time trends were not controlled for. Finally, our results varied only slightly when the classification in HIC and MIC categories was based on the modal World Bank classification value observed over the period 1987–2010, leading to reclassify 13 countries as MIC (eg, Argentina, Chile, Russia). The only significant change was that international trade was also associated to an increase in the share of sugar intake for MIC over the early period 1970–1989.

## Discussion

This analysis indicates that upwards trends in food innovations are significantly associated with upwards trends in calorie supply, and with changes in the macronutrient composition of the diet. Most notably, food innovations had significant, positive and large associations with calorie supply in MIC over 1990–2010, a period of intense integration of these countries within the world economy. A positive and significant association was also observed between innovations and the share of animal and free fats in the total food supply between 1970 and 1989 for MIC and between 1990 and 2010 for HIC. Finally, the diffusion of food innovations was associated with the substitution of complex carbohydrates for sugars in the composition of total food supply. This pattern was especially strong for MIC in the second period (1990–2010). For HIC, these results were explained by direct innovations, that is, patent applications in these countries, while imports of processed food products incorporating innovations were decisive for explaining these trends in MIC. Importantly, all results were robust to the inclusion of control variables measuring confounding demand factors, such as income per capita, women labour force participation, urbanisation rate, social and economic globalisation, country-specific factors (eg, food culture) and income-group specific time shocks.

These results shed light on the role of alterations in the food supply on the obesity epidemic, particularly through the increasing international production and diffusion of ultra-processed foods.

First, our findings complement the existing literature showing significant associations between changes in the technical and nutritional characteristics of food products, and changes in the nutritional health status of populations, in particular in MIC.^10 26 27^ The NOVA classification, frequently used to measure these technical characteristics and identify ultra-processed foods, is sometimes discussed.^28 29^ In this study, we abstract from these discussions by directly measuring the international diffusion of food innovations using patent data. The descriptive analyses show that these innovations are increasingly concentrated in one class of the PATSTAT nomenclature that includes most of the processes playing a role in the production of ultra-processed foods (online supplemental appendix p 16).

Second, the results suggest nuancing statements about the effect of supply factors on food systems and nutritional health. Figure 1 shows significant raw correlations between food innovations and four markers of the nutritional transition: the share of complex carbohydrates declines with the stock of innovations, while the shares of sugars, fats and animal proteins increase. Yet, table 2 indicates that these results are robust for sugars, complex carbohydrates and fats, consistent with studies highlighting the contribution of the diffusion of sugar-sweetened food and beverages to the obesity epidemic in MIC.^30^ However, for animal proteins, the raw correlations are attenuated and become almost not significant in multivariate regressions when controlling for confounding trends in demand factors. Hence, the increasing importance of animal proteins in the diets is mainly driven by demand-side components rather than supply-side components. This result is consistent with previous studies underlying the role of food demand changes in obesity, declining cooking time and reliance on processed or prepared, or social and cultural globalisation.^1 16 19 31^

Our study is the first to use patent data to examine the relationships between technological innovations and nutritional quality of the food supply with country-year level data. The results show the complexity of interactions that are heterogeneous across countries’ income groups, time periods and nutritional outcomes. However, our study has several limitations. First, patent data are imperfect measures of technological innovation (online supplemental appendix pp 1–2). The decision to file a patent application in a country depends on many institutional characteristics and expected market effects. Alternative measures could be envisioned, such as expenditures in research and development in food industries. Unfortunately, these data are not easily available for a large panel of countries and years. Moreover, the increase in supply incorporating more technology is accompanied by marketing efforts that may also alter consumer preferences. Our empirical strategy only imperfectly controls for these aspects by adding country and year fixed effects, and income group specific year effects. Second, technological innovations can affect the overall nutritional composition of the food supply by changing the composition of products and by changing the relative prices of products. We were not able to separate these mechanisms. Third, technological innovations in food production eased the development of mass retailing and fuelled the rise of supermarkets, a major channel linking innovations to the decline in money and time costs of meal preparation. In the absence of harmonised statistics on food retailing, we were not able to document the mediating role of supermarkets expansion.

## Conclusion

This is the largest multicountry study using representative innovation data for 38 HIC and 29 MIC to analyse the international diffusion of food innovations across countries and over years. Two subperiods were distinguished: 1970–1989 and 1990–2010, the latter being marked by the hyperglobalisation of economies and food supply chains. We provided the first estimation of the association between trends in innovations and indicators of the nutrition transition, after adjusting for the confounding effects of demand-side pulling factors. In general, we evidenced a positive and significant correlation between innovations and the share of sugars in total food supply and a negative correlation for the share of complex carbohydrates. We also highlighted a positive and significant association between innovations and the share of fats in total food supply, mainly driven by animal and free fats. In addition, we observed a large positive and significant correlation between innovations and the share of sugars in total food supply in MIC for the period 1990–2010. For this group of countries, the trade channel capturing the access to innovations through imports of processed food products was decisive for understanding the nutrition transition patterns.

These results can contribute to the design of effective public policies to promote healthier diets and reduce overweight and obesity prevalence. They show that a better interaction between public health and trade policies is crucial. We uncovered quantitative evidence that innovations have significantly transformed food supply and its main components, especially sugars, beyond changes driven by consumer demand. New food technologies are directly diffused worldwide by inventors requesting protection in different countries, but also through international trade of processed food products encapsulating these technologies. In the current regulation framework for international trade, countries can enforce sanitary and phytosanitary measures—potentially restricting imports from foreign countries—to ensure the safety of food products available on their markets. However, up to date, measures dealing with the nutritional quality of food products are not allowed. Our results highlight that public policies could be more directly targeted at certain production technologies and innovations, for example, through quality standards. Sugar is a good example of potential deeper public intervention. As shown in our analysis, supply-side effects impact significantly the share of sugars in the total food supply.

Yet, this targeting must also consider the existence of a genuine consumer demand for ultra-processed food, which is not only highly palatable, but also easy to use and requires no preparation time. Future work should further enrich food product databases with accurate information on the technologies used in their production, in order to document more precisely the causal chain linking innovations, food products and health risks. Furthermore, in line with the initiative ‘One world, one health’, one could also go one step further and investigate the impact of food innovations on health outcomes (eg, obesity prevalence, cardiovascular diseases and diabetes)

Finally, our study investigates the international diffusion of food innovations. A natural extension—relying on additional data—would consist in the exploration of the heterogeneity in the diffusion of innovations within countries. Such approach will help understanding nutritional inequalities within countries and identifying socioeconomic groups most exposed to the effects of food innovations. Besides, we restricted our focus on HIC and MIC, which receive most of innovations. Research on low-income countries—which often suffer from double burden malnutrition—may also be useful and provide a better comprehension of the mechanisms at play in these countries.

## Data Availability

The FAO on food supply and country-level characteristics are publicly available and downloadable from https://www.fao.org/faostat/en/ The World Bank data on country-level characteristics are publicly available and can be downloaded from https://databank.worldbank.org/ The KOF data on globalisation are publicly available data and can be downloaded from https://kof.ethz.ch/en/forecasts-and-indicators/indicators/kof-globalisation-index.html The PATSTAT data are commercial data that can be purchased on the website of the European Patent Office (https://www.epo.org/searching-for-patents/business/patstat_fr.html).
