# Supplementary material for: The international diffusion of food innovations and the nutrition transition: retrospective longitudinal evidence from country-level data, 1970–2010
Source: BMJ Glob Health. 2023 Nov 15;8(11):e012062. doi: 10.1136/bmjgh-2023-012062 (PMC10660632; doi:10.1136/bmjgh-2023-012062)
Supplement: Supplementary data [file bmjgh-2023-012062supp001.pdf]

The international diffusion of food innovations and the nutrition transition: retrospective longitudinal evidence from country-level data, 1970-2010.

Anne-Célia Disdier[a,b], Fabrice Etilé[b,a]

Lorenzo Rotunno[c]

[a]Paris School of Economics, F-75014 Paris, France

[b] INRAE, UMR 1393 PjSE, F-75014 Paris, France

[c]Aix-Marseille School of Economics, France

Supplementary Appendix

This version: May 31, 2023

A Data

A.1 The patenting process

A patent intends to protect a process or a product. A company or a person that wants to protect an innovation in a given country has to file an application with a patent authority that acts at a national level (e.g. the USPTO, United States Patent and Trademark Office) or at a regional level (e.g the EPO, European Patent Office). Filing an application does neither mean that the patent will effectively be granted nor allow the applicant to sell the rights of using the innovation. Filing a patent application prevents the exploitation of the innovation by a third-party in a set of designated states during the entire examination process. The choice of specific designated states and patent authorities depends on various strategic motives. The applicant may want to use the innovation and to benefit from a monopolistic position over the technology in specific states. The applicant may want to pre-empt a technology to block competitors, even in countries where it does not operate. The applicant may also choose patent authorities depending on its expectations regarding the success of the examination procedure.

Before granting a patent, a patent office often produces a report evaluating the patentability of the invention, but this is not always the case. Three cases can be distinguished. First, a patent can be granted with simple formality examination, in which case patenting is easy. However, the patent owner has no information about the legal risks that a third-party files a request for nullification with a court. Second, a patent can be granted after formality examination *and* prior art search. The application is costlier, but the applicant gains awareness of the potential legal risks. However, third-parties can also better assess the validity of the granted patent upon its publication. Third, a patent can be granted after formality examination, prior art search and substantive examination. Skilled examiners carefully review and evaluate the patentability of the invention. The process is even costlier, but ex post litigation risks are largely reduced. As substantive examination entails important human and financial costs, patent offices and states exchange the results of search and examination works within the framework of the Patent Cooperation Treaty (PCT). When a patent application is first filed at a national or regional patent office as a PCT application, it becomes an international application. Otherwise, it is a regional or a national application, which restricts the set of designated states where the invention is likely to be protected.

Each patent application is associated to a *priority date*. The date of priority is the effective date of filing for examination of the inventive step of the first application and all the subsequent applications claiming the priority of the first application. The priority date determines the date until which prior art is taken into account for search examination. At an international level, the convention priority right is regulated by the Paris Convention of 1883. Once the priority date is determined, the delay for the publication of the application at a national level can vary from 18 months for a first

national or regional application to 48 months for PCT applications.[1] Some patent offices, such as the USPTO, do not automatically publish the application. Importantly, a patent can be granted several years after the priority date.

A.2 Patent data

The PATSTAT[2] data include information on tens of millions of patent applications, such as the legal links between patent applications, with which authority application has been filed, when it was filed (its priority date), the collection of legal events that characterised the application process, etc. An application is registered in PATSTAT if it has at least one publication. It is otherwise regarded as confidential. The applications are linked through their priority dates. Multiple priorities can be claimed by an application to cover different parts of the invention. Several applications covering the same invention can also claim the same priority date. This happens when the applicant files applications with different offices, or when the applicant files, within a one-year period, a new application that slightly modify the previous one. This network of cross-references and priority dates defines an extended family (or INPADOC family) wherein applications have technical and continuation relations. In the data construction process, our time index was the priority date, and we chose the earliest priority date for applications that had several priority dates. Our results are robust to the selection of alternative priority dates for multi-date applications.

The construction of patent count variables required to make choices regarding the selection of patents. Our main variable of interest was the country in which an (domestic or foreign) inventor seeks for protection.

We first selected patents related to food processing. Patent applications are assigned International Patent Classification (IPC) codes by patent offices. We picked the subset of patent applications with 100% of IPC codes related to food processing (Supplementary Table 1). In our sample, about 70% of applications were filed by firms.

We did not restrict the sample to patent applications that were eventually successful (granted patents), but to all patent applications that were filed or under examination over the period of interest, from 1970 to 2010. We decided not to use the data after 2011 included in the PATSTAT database acquired in 2018, because of a sharp drop in the number of applications. This can be explained by the data collection process. The publication of applications can take up to 48 months and some countries appear to transmit information with important delays. The choice of including also patent applications that eventually did not lead to patent grants was motivated by time or cross-country variability in patent evaluation processes. A given innovation may indeed be successfully patented by one authority but not another, because different patent authorities may retain different criteria for patent evaluation, or a patent authority changed its examination process. In addition, the duration of the examination process varies a lot across authorities.[3] But an innovation is protected once a patent application is filed. This implies that application filing dates are likely to better reflect the use of applications by firms.

Yet, the decision to file an application is a strategic decision for firms.(4) They may decide not to file to keep the innovation secret. However, the most innovating firms patent more frequently than followers, patenting activity is positively correlated with profits, and granted patents are associated with economically significant inventions.(5,6)

Firms may also file applications not for using them but with the strategic aim of blocking innovations by competing firms. To limit concerns with this kind of strategic use of patents, we followed the literature and decided to restrict the sample to *patent families* that appeared to be the most important according to the patent authority.(7) Patent families are applications that are connected through a network of cross-references, meaning that they refer to the same

---

[1] An application that is filed at an international level (as a PCT application) will, after examination, enter in a regional or national examination phase except if the applicant decides to abandon.
[2] Past research has often used data from the US Patent Office (USPTO), but it tends to over-represent North-American companies and, until 2011, disclosed statistics on patents granted only. Hence, the USPTO data would not suit our purposes. See de Rassenfosse and colleagues (2013, 2014) for an introduction to the Patstat database.(1,2)
[3] For instance, international applications under the Patent Cooperation Treaty can be filed with the Japan Patent Office within a delay of 30 months from the priority date or the earliest filing date, and then "the application may not be examined unless the applicant (or a third party) makes a written request within a period of seven years from the date of filing in Japan" in The Thomson Corporation (2007, p. 114).(3)

fundamental innovation.[4] We tagged patent families with applications filed at least in the two most important patenting authorities. This makes it more likely that these patent families are based on a higher-valued invention and that the firm expects to commercialize the invention (4). Studies of innovating activities by countries generally focus on a small set of patenting authorities, i.e. the triad Europe, US and Japan, plus sometimes South Korea (7). We extended the list to include countries that displayed a significant number of inventors over the period. The list therefore includes economies that have grown rapidly or that have played an important role in the dynamics of food systems over the last decades, such as Mexico or China.

We thus applied the following sequence of decisions (see Supplementary Figure 1 for counts of selected observations at each step):

1. Select the subsample of invention-related patent applications with at least one food processing IPC code (as primary code when the latter is designated by the patent authority); Drop artificial patent applications;[5] Keep applications filed after 1950; Keep Intellectual Property Type related applications.
2. Drop applications that have less than 100% of food processing IPC codes; Drop applications that are neither in national or regional phase; Tag INPADOC families with applications filed at least with two of the following patenting authorities:[6] Australia, Brazil, Canada, China, Europe (EP), France, Germany, Great Britain, Italy, Japan, South Korea, Mexico, Netherlands, Russia/Soviet-Union, South-Africa, Spain, Switzerland, USA.
3. Disaggregate regional-level applications to assign them to countries, using the presence of any positive legal event as evidence that the patent has some value. We used the information on legal events that PATSTAT recodes as positive or negative. These legal events are often associated to specific countries. For instance, an applicant files an application with the European Patent Office but chooses to designate only a subset of European states. We also dropped the applications without at least one industry NACE code relating to the manufacturing of food products or beverages.

We eventually obtained for each country c a count of new major patent applications in year t: $NEWPATENTAPPLICATIONS_{ct}$. Major applications are those in tagged INPADOC families (Step 2). Patent applications count can be constructed for all IPC codes listed in Supplementary Table 1 or for selected groups of IPC codes (see Supplementary Figure 5).

A.3 Other data

A.3.1 FAO data on food and nutrition

We used the food balances data from the Food and Agriculture Organization of the United Nations (FAO) to derive our key indicators of changes in food supply over the period 1970-2010. The FAO provides measures of total food supply in kcal per capita per day and protein supply and fat supply in gram per capita per day. Protein supply and fat supply were converted into kcal per capita per day by assuming that one gram of protein provides 4 kcal and one gram of fat provides 9 kcal. The residual calorie supply, obtained by subtracting proteins and fats from the total supply, is provided by carbohydrates and sugars.

We then distinguished between vegetable and animal protein sources, sugars versus complex carbohydrates, and animal or free fats vs. vegetable fats. Free fats are defined as fats that have been extracted from an animal or vegetable product: all varieties of oils, butter, etc. To construct these measures, we combined the FAO data at the level of food items with

[4] We chose to work with PATSTAT INPADOC families. INPADOC families are disjoint sets of applications. All applications grouped in a same INPADOC family are related either directly or indirectly through priority dates.
[5] An application that has been abandoned by the applicant is not published, but PATSTAT refers to it as an artificial application when it is cited by a published application.
[6] The OECD recommends to identify valuable innovations by focusing on triadic patent families. These are the patent families filed at both the EPO and the JPO and granted by the USPTO (8). We did not apply this criterion because the USPTO can have lags of several years between the date of application filling and the granting date, see De Rassenfosse and colleagues for a thorough discussion (1). In addition, we wanted to include food companies from main emerging economies.

our own classification of food items as providing one or another category of nutrient. For instance, the FAO information fat supply provided by oils is entirely assigned to the category of free or animal fats (see Supplementary Table 2, pp 21-22). We then aggregated this information to calculate the *share* of total calorie supply provided by sugars, complex carbohydrates, free or animal fats, vegetable fats, animal proteins, vegetable proteins.

We also computed a measure of the share of imported calories in total calorie supply as:

- $SHAREIMPORTFOOD_{ct} =$
  $\max\left(0, \min\left(\frac{(IMPORTSUPPLY_{ct} - EXPORTSUPP\quad_{ct}) \times (1 - STOCKSUPPLY_{ct}/(DOMESTICSUPPLY_{ct} + STOCKSUPPLY_{ct}))}{DOMESTICSUPPLY_{ct}}, 1\right)\right)$
  if $STOCKSUPPLY_{ct} \geq 0$
- $SHAREIMPORTFOOD_{ct} = \max\left(0, \min\left(\frac{(IMPORTSUPPLY_{ct} - EXPORTSUPPLY_{ct})}{DOMESTICSUPPLY_{ct}}, 1\right)\right)$
  if $STOCKSUPPLY_{ct} < 0$

where we used FAO data on imported calories ($IMPORTSUPPLY_{ct}$), exported calories ($EXPORTSUPPLY_{ct}$), total food supply ($DOMESTICSUPPLY_{ct}$) and stocks ($STOCKSUPPLY_{ct}$). For negative stocks, we assumed that the difference between imports and exports gives the share of total food supply that is provided by imports (with left- and right-truncations at 0 and 1). This tends to underestimate the share of imports in total food supply: an extreme example would be a country that would import all its food for domestic consumption and would export all its agri-food production. For positive stocks, we assumed that part of the difference between imports and exports had been allocated to stocks, so that only a fraction remained available for domestic consumption:

$(1 - STOCKSUPPLY_{ct}/(DOMESTICSUPPLY_{ct} + STOCKSUPPLY_{ct}))$.

A.3.2 UN Comtrade trade data

We used the United Nation Comtrade dataset to construct a measure of innovations embedded in the processed food imported by a country *c* from a country *c'*. As we need data covering the 1970-2010 period, we relied the Comtrade database with the Standard International Trade Classification of commodities (SITC, rev. 1). The Comtrade database provides import flows by origin-destination-SITC category-year. We recovered the share of each country *c'* in the total import value of processed food for country *c* (in US $).

In the SITC classification, we retained the following 4-digit categories of commodities for defining processed food : meat extract (0133), sausages (0134), other prepared or preserved meat (0138), fish in airtight container (0320), macaroni/spaghetti/noodles/etc. (0483), bakery products (0484), preparation of cereals/flour/starch (0488), fruit preserved by sugar (0532), jams etc. (0533), fruit juices (0535), fruits preserved or prepared (0535), vegetables preserved or prepared (0539 and 0555), raw sugar (0611), refined sugar (0612), molasses (0615), sugar syrups etc. (0619), sugar confectionery (0620), coffee extracts (0713), cocoa products (0721, 0722, 0723), chocolate products (0740), food preparations (0990).

A.3.3 Data for country-level socio-economic indicators

We used the World Bank dataset of World Development Indicators (WDI) to construct country-year level measures of income per capita, female labour force participation, and urbanization. These data are available at https://databank.worldbank.org/source/world-development-indicators. They are made up of information collected by various international bodies.

Income per capita is measured as the annual Gross Domestic Product (GDP) divided by population size, in constant 2010$. It is calculated by the World Bank, using World Bank and OECD national accounts data.

Female labour force participation is measured as the labour force participation rate of females in % of the working-age female population, where the definition of the latter accounts for cross-country sources of variability such as minimum and maximum legal working ages. It is provided by the International Labour Organisation. Data for missing years in country series were completed by linear interpolation.

Urbanisation is measured as the proportion of the total population residing in an urban area as defined by national statistics offices. It is calculated by the World Bank, using population estimates and urban ratios from the United Nations World Urbanization Prospects.

Finally, we used the 2014 World Bank classification of countries by income level to distinguish high-income countries (HIC) from upper middle-income countries (UMIC), lower middle-income countries (LMIC), and low-income countries (LIC). UMIC and LMIC are merged into the group of middle-income countries (MIC). The groups change over time because of changes in the thresholds adopted by the World Bank, and because of variation in the Gross National Income (GNI) of the countries. In our analysis, we fixed the income groups to the 2014 classification to avoid overlapping of the MIC and HIC samples – this would have happened if the same country appeared in one group for some years and in the other groups in another period. We did not choose 2010 to avoid the noise produced by the Great Recession of 2008-2010. In a sensitivity analysis, we adopted an alternative approach and assigned each country to its most recurrent income group between 1987 (the World Bank income classification starts in 1987) and 2010. With this alternative definition, thirteen countries were moved from the HIC group to the MIC group: Argentina, Chile, Croatia, Czech Republic, Estonia, Hungary, Latvia, Lithuania, Poland, Saudi Arabia, Slovak Republic, Uruguay, and the Russian federation.

A.3.4 Globalization indices

The KOF Swiss Economic Institute provides multidimensional measures of globalization (9,10). Following our past work, we used an index of economic globalization and an index of social globalization (11). This study exploits the 2019 version of the dataset, downloaded from https://kof.ethz.ch/en/forecasts-and-indicators/indicators/kof-globalization-index.html.

Economic globalization measures the extent of trade and financial embeddedness of a country in the world economy, as reflected by the exports and imports of goods and services, the diversity of trade partners, foreign direct investments, portfolio investments, and international debt, reserves and income payments.

Social globalization focuses more on the extent to which the country population has contact with foreign populations, cultures and lifestyles. It aggregates indicators for interpersonal relationships (international voice traffic, transfers, international tourism, migration, international students), informational globalization (used internet bandwidth, high technology exports), and cultural globalization (trade in cultural goods and personal services, international trademarks, McDonald's restaurants, IKEA stores).

Both indexes vary between 1 and 100, where 100 represents the maximum value.

A.4 Steps in sample selection and country-year matching

We started from the final dataset of patent counts obtained in Section A.2. This dataset included 89 countries among which 85 have at least one major application in a given year between 1970 and 2010. We thus ignored countries that are either not covered by the PATSTAT data collection system, or that are covered but are not designated in food-related patent applications of significant importance. The rationale is that these countries are unsuitable for identifying the association between patent applications and nutritional outcomes, either because they have specific unobserved characteristics (institutions, economy) that can simultaneously affect their participation to the international patenting system and their food markets, or because they do not represent food markets of significant importance for agri-food companies.

We then applied the following exclusion/inclusion rules:

- Drop former Eastern European countries for which we had some patent information, but no trade information or World Bank data: Czechoslovakia, The German Democratic Republic, Soviet Union, Yugoslavia. We dropped Serbia, Montenegro due to the absence of trade data, even for the most recent years.
- Drop Taiwan (World Bank Data missing).
- Drop countries that are observed less than 16 years, such as Cuba, Dominican Republic, Monaco, Singapore, and Tajikistan. This threshold was chosen to keep a maximum number of countries in the sample over a

maximum number of observation periods. Increasing the threshold results in the loss of the many countries that became members of the PCT in the 90s (e.g., the Eastern European countries).

- For the following former Central and Eastern European countries and South Africa, drop observations before 1992: Bulgaria, Croatia, Estonia, Georgia, Hungary, Lithuania, Moldovia, Poland, Romania, Russia, Ukraine, South Africa.
- For some countries, keep observations after a certain year on a case-by-case basis, depending on the availability and continuity of data series: China (1988-2010), Czech Republic (1994-2010), Slovakia (1994-2010), Slovenia (1995-2010), Viet-Nam (1997-2010), Zambia (1995-2010).
- Drop countries with less than one million inhabitants (census data) on average over the period of observation: Cyprus, Iceland, Malta.
- Drop low-income countries: Malawi, Zimbabwe.

We ended up with an unbalanced sample of 67 countries, including 38 HIC and 29 MIC (see Supplementary Figures 2 and 3). The balanced panel includes 43 countries: 25 HIC and 18 MIC. We decided to present estimation results from the unbalanced sample, to keep important countries such as China in the analysis. We used the balanced sample for sensitivity analysis.

Supplementary appendix A, references

1. de Rassenfosse G, Dernis H, Guellec D, Picci L, van Pottelsberghe de la Potterie B. The worldwide count of priority patents: A new indicator of inventive activity. Research Policy. 2013;42(3):720–37.

2. de Rassenfosse G, Dernis H, Boedt G. An introduction to the Patstat database with example queries. Australian Economic Review. 2014;47(3):395–408.

3. The Thomson Corporation. Global Patent Sources: An Overview of International Patents. London: Thomson Scientific; 2007.

4. Kleinknecht A, Van Montfort K, Brouwer E. The Non-Trivial Choice between Innovation Indicators. Economics of Innovation and New Technology. 2002;11(2):109–21.

5. Dernis H, Guellec D, van Pottelsberghe, Bruno. Using patent counts for cross-country comparisons of technology output. OECD STI Review. 2001;27:127–46.

6. Hanel P. The Use of Intellectual Property Rights and Innovation by Manufacturing Firms in Canada. Economics of Innovation and New Technology. 2008;17(4):285–309.

7. Dernis H, Khan M. Triadic Patent Families Methodology [Internet]. Paris: OECD; 2004 [cited 2023 Feb 2]. (OECD Science, Technology and Industry Working Papers). Report No.: 2004/02. Available from: https://www.oecd-ilibrary.org/science-and-technology/triadic-patent-families-methodology_443844125004

8. OECD. OECD Patent Statistics Manual 2009. Paris: OECD; 2009.

9. Dreher A. Does globalization affect growth? evidence from a new index of globalization. Applied Economics. 2006;38(10):1091–110.

10. Gygli S, Haelg F, Potrafke N, Sturm JE. The KOF globalization index – revisited. The Review of International Organizations. 2019;14(3):543–74.

11. Oberlander L, Disdier A, Etilé F. Globalization and national trends in nutrition and health: A grouped fixed-effects approach to intercountry heterogeneity. Health Economics. 2017;26(9):1146–61.

B Data analysis

B.1 Construction of the measure of food innovations

We first computed a measure of domestic food innovations in country *c* in year *t*, $\mathrm{PATENTSTOCK}_{ct}$, as the *stock* of patent applications designating this country:

$$PATENTSTOCK_{ct} = (1-\delta)PATENTSTOCK_{ct-1} + NEWPATENTAPPLICATIONS_{ct}$$

where δ is a parameter that may account for the depreciation of innovations and the stock starts at 0 in 1950. Our main results used a patent stock variable constructed with $\delta = 0$, meaning that technological progress is an irreversible accumulation process. In a sensistivity analysis, we produced results with $\delta = 5\%$ because, in many jurisdictions, patents protect innovations during 20 years. Hence, as a first-order approximation, it loses its market value after 20 years (for the same reason, the stock is built from 1950, as the period of analysis starts in 1970). The regression results were left unaltered, essentially because country fixed effects were included in all regressions: identification was based on the association between changes in food technology diffusion and changes in nutritional patterns, where changes in technology diffusion essentially depended on new patent applications.

We then computed the annual stock of food innovations present in each country, $\mathrm{INNOVSTOCK}_{ct}$, as a weighted sum of the stock of patent applications designating this country ($\mathrm{PATENTSTOCK}_{ct}$) and the stock of patents indirectly obtained through the imports of processed food products. We decided to focus on processed food products as such products are more likely to incorporate food innovations. We used the following formula:

$$INNOVSTOCK_{ct} = (1 - SHAREIMPORTFOOD_{ct}) \times PATENTSTOCK_{ct}$$

$$+SHAREIMPORTFOOD_{ct} \times \sum_{c\prime \neq c}(SHAREIMPORTPROCESS_{cc\prime t} \times PATENTSTOCK_{c\prime t})$$

where

- $SHAREIMPORTFOOD_{ct}$ is the share of imports in total calorie supply for human consumption of country *c* in year *t*, calculated from FAO data on stocks and flows (imports, exports, domestic production) of calories (see Section A.3.1 of this Supplementary Appendix).
- $SHAREIMPORTPROCESS_{cc\prime t}$ is the share of country c' in import value of processed food by country *c* in year *t*, calculated from the COMTRADE/SITC data (see Section A.3.2 of this Supplementary Appendix). The volume of food technology "imported" by country *c* in a given year is then a weighted average of the food technology produced by all its trading partners *c'*, where the weights equal the import shares and food technology is measured by the patent stock of each trading partner.

B.2 Statistical analyses

To estimate the associations between nutritional outcomes and food innovations, we relied on regression models, where total calorie supply and the share of this supply provided by specific macro-nutrients were the dependent variables and our measure of innovation, $INNOVSTOCK_{\mathrm{ct}}$, was the main covariate of interest.

Our analysis covered the period 1970-2010. To capture potential changes in the associations over time, we added an interaction term between $INNOVSTOCK_{\mathrm{ct}}$ and a dummy DECADES that equals 1 for years *t* in 1990-2010 (0 otherwise). Years 1990-2010 are characterized by an acceleration of the globalization of countries.

We ran separate regressions for HIC and MIC. The model was adjusted for country-specific factors that can be interpreted as demand-side drivers of the dynamics of food markets (see Section 3.2 in the main text): Gross Domestic Product (GDP) per capita, women labour force participation (WLFP), urbanization rate, and an indicator of social globalization. To capture some potential non-linearities, we also included the squared GDP per capita and the squared WLFP. We further adjusted for economic globalization and the share of food imports in total calorie supply, as these variables are likely to have a direct effect on nutritional outcomes, beyond their indirect effect through the food innovation measure. We also controlled for whether the country is a member of the Patent Cooperation Treaty (PCT) and/or a member of the Paris Convention, as these memberships influence the patent application counts (see Section A.1 of this Supplementary Appendix).

We included country fixed-effects, and year fixed-effects. When estimating the model on MIC, the year fixed-effects were further interacted with income-group fixed effects distinguishing UMIC from LMIC. We thus measured the correlation between within-country changes in the total stock of food innovations and in nutritional outcomes conditional on within-country variations in the covariates and income-group specific year shocks. The model was estimated using Stata 17.0 command reghdfe and standard errors were clustered by country. The main specification is:

$$ln(Y_{ct}) = \alpha_0 ln(INNOVSTOCK_{ct}) + \alpha_1 ln(INNOVSTOCK_{ct}) \times DECADES_{ct} + \beta X_{ct} + \delta_c + \delta_{g_c t} + \epsilon_{ct} \quad \textbf{(Equation 1)}$$

where:

- ln(.) is the neperian logarithmic function
- Y_{ct} is total calorie supply or the shares of macro-nutrients. For these shares, we applied a logit transformation, so that $Y_{ct} = NUTRIENTSHARE_{ct}/(1 - NUTRIENTSHARE_{ct})$. This allows a better fit of the data given the shape of trends in nutrient shares (see Figure 1).
- $INNOVSTOCK_{ct}$ is the annual stock of food innovations
- $DECADES_{ct}$ is a dummy set to 1 for years 1990-2010 (0 otherwise)
- X_{ct} is a vector of controls (GDP per capita, squared GDP per capita, women labour force participation, squared women labour force participation, urbanization rate, social globalization, economic globalization, share of food imports in total calorie supply, membership to PCT and/or Paris Convention
- δ_c are country fixed-effects
- $\delta_{g_c t}$ are country income-group x year fixed-effects
- ϵ_{ct} is the error term

B.3 Computation of elasticities and counterfactuals

Elasticities: Tables 2 (main text) and Supplementary Tables 5 and 6 (Supplementary Appendix) report elasticities instead of estimated coefficients. An elasticity measures the responsiveness of a variable to an increase in another variable. For instance, in Equation (1), α is the elasticity of Y_{ct} to $INNOVSTOCK_{ct}$. Say differently, it is the relative increase in Y_{ct} (in %) that is associated to a 1% increase in $INNOVSTOCK_{ct}$. For total food supply, α can be directly interpreted as an elasticity since we have a log-log relationship (this uses the approximation $\frac{\Delta Y}{Y} \Big/ \frac{\Delta X}{X} \approx \partial log(Y) \big/ \partial log(X)$ for small changes in X). For the nutrient shares ($NUTRIENTSHARE_{ct}$), given the logit transformation, we reported an elasticity calculated at the mean of the estimation sample:

$$Elasticity_{gd} = (1 - \overline{NUTRIENTSHARE_{ct}}^{gd})(\alpha_0 + \alpha_1 \times d)$$

where $\overline{NUTRIENTSHARE_{ct}}^{gd}$ is the average nutrient share for countries in income group *g* (HIC or MIC) observed in decades 1970-1989 (*d*=0) or 1990-2010 (*d*=1).

Counterfactuals: Figures 2 and 3 (main text) were produced by comparing the actual situation with a counterfactual situation, obtained either by setting:

- The stock of food innovations at the technological frontier (i.e. the maximum stock observed in the same income-group and same period) and assuming PCT and Paris Convention memberships (Figure 2)
- The stock of innovations to the value it would have if innovations only arrived directly from inventors seeking protection in the territory and not also indirectly through imports of processed food products (i.e. setting artificially $SHAREIMPORTFOOD_{ct}$ to 0 in the formula of Section B.1, as if $INNOVSTOCK_{ct} = PATENTSTOCK_{ct}$).

Then, let Y^1 be the counterfactual value of Y, and Y^0 its actual value. For the first counterfactual (technological frontier), we computed a relative variation in Y by income group *g* and period *d* for the (hypothetical) average country as:

$$\frac{Y^1 - Y^0}{Y^0} = exp\left((\alpha_0 + \alpha_1 \times d) \times \left(Max_{g,d}(INNOVSTOCK_{ct}) - \overline{INNOVSTOCK_{ct}}^{g,d}\right) + \beta_{PCT} \times \left(1 - \overline{PCT_{ct}}^{g,d}\right) + \beta_{PARIS\ Conv.} \times \left(1 - \overline{PARIS\ Conv._{ct}}^{g,d}\right)\right)$$

while for the second counterfactual (without imported food innovations), the expression is:

$$\frac{Y^1 - Y^0}{Y^0} = exp\left((\alpha_0 + \alpha_1 \times d) \times \left(\overline{INNOVSTOCK_{ct}}^{g,d} - \overline{PATENTSTOCK_{ct}}^{g,d}\right)\right)$$

where:

- d equals 1 if one computes the counterfactual for period 1990-2010
- g is an index distinguishing HIC from MIC
- $\overline{X_{ct}}^{g,d}$ is the average of X for all observations of country *c* in income-group *g* and years in period 1970-1989 (if *d*=0) or 1990-2010 (if *d*=1)
- $Max_{g,d}(INNOVSTOCK_{ct})$ is the maximum value of $INNOVSTOCK_{ct}$ in the set of countries *c* in income-group *g* and years in period 1970-1989 (if *d*=0) or 1990-2010 (if *d*=1)

For total food supply, these formulas directly provide the quantity of interest. For nutrient shares, as we used a logit transformation for the regressions, we applied a further transformation. Let $\frac{Y^1-Y^0}{Y^0} = \Delta^c$:

$$\frac{NUTRIENTSHARE^1 - NUTRIENTSHARE^0}{NUTRIENTSHARE^0} = \frac{\Delta^c \times \left(1 - \overline{NUTRIENTSHARE_{ct}}^{g,d}\right)}{1 + \Delta^c \times \overline{NUTRIENTSHARE_{ct}}^{g,d}}$$

Sensitivity analysis: For the first counterfactual (technological frontier), we also computed the counterfactual variations without accounting for PCT and Paris Convention memberships as:

$$\frac{Y^1 - Y^0}{Y^0} = exp\left((\alpha_0 + \alpha_1 \times d) \times \left(Max_{g,d}(INNOVSTOCK_{ct}) - \overline{INNOVSTOCK_{ct}}^{g,d}\right)\right)$$

Results with this alternative formula are reported in Supplementary Figure 6.

Point estimates and standard errors for the elasticities and the counterfactual variations were all obtained by using the Stata post-estimation commands *nlcom* and *margins*.

C Supplementary figures and tables

**PATSTAT patent applications:**<br>N = 91,738,066 applications

**Step 1:**<br>N = 932,500 applications<br>104 application authorities	Step 1: Keep applications with at least one food processing IPC code; Drop artificial patent applications; Keep applications filed after 1950; Keep Intellectual Property Type related applications.
**Step 2:**<br>N = 723,678 applications<br>93 application authorities	Step 2: Keep applications with only food-related IPC codes, and that have entered the regional or national phase; Keep major applications.
**Step 3:**<br>N = 705,718 applications<br>89 designated states<br>90 authorities	Step 3: Disaggregate regional-level applications and merge information on designated countries; Keep only applications that have at least one industry NACE code related to the manufacturing of food products or beverages; Keep only applications with at least one positive legal event.

PATENT datasets:<br>N = 705,718 applications at 90 authorities with 89 designated countries<br>N = 184,776 major applications at 86 authorities with 85 designated countries<br>Years = 1970-2010

Supplementary Figure 1 – Patent data, selection rules

PATENT sample:<br>N = 85 designated countries<br>Years = 1970-2010

Step 1<br>N = 79 authorities<br>Years = 1970-2010	Exclusion rule 1: Drop former Eastern European countries.
Step 2:<br>N = 72 designated countries<br>Years = 1970-2010	Exclusion rule 2: Drop countries not in the UN Comtrade and WDI datasets and countries observed less than 16 years.
Step 3:<br>N = 72 designated countries<br>Years = 1970-2010	Exclusion/inclusion rule 3: For several Central and Eastern European countries and South Africa, drop observations before 1992; For some other countries, keep observations after a certain year.
Step 4:<br>N = 67 designated countries<br>Years = 1970-2010	Exclusion rule 4: Drop countries with less than one million inhabitants and low-income countries.

Final sample:<br>N = 67 countries (38 HIC and 29 MIC)<br>Years = 1970-2010

Supplementary Figure 2 – Final sample, selection rules

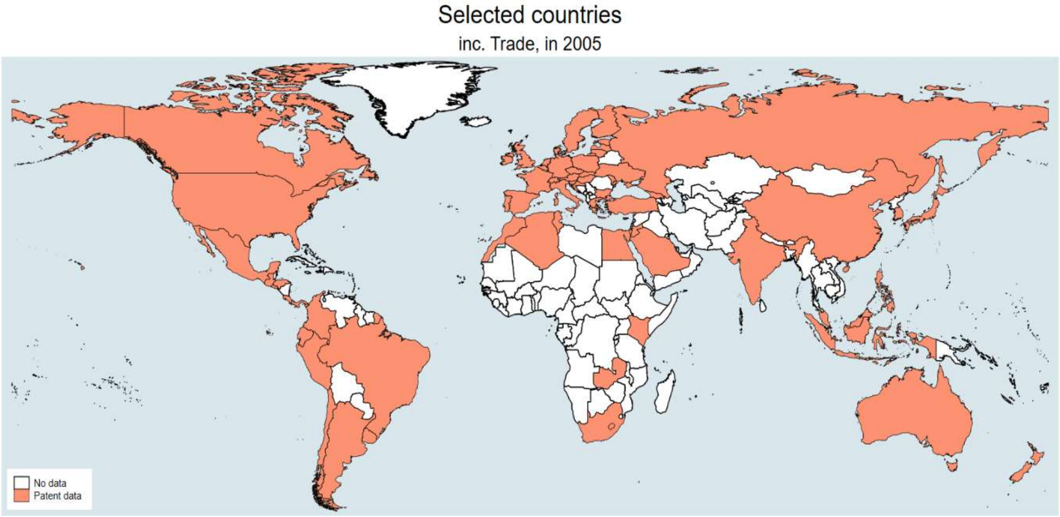

Supplementary Figure 3 – Countries included in the sample in 2005

Note: List of countries (World Bank, 2014 classification):

- Lower Middle-Income Countries: Egypt, El Salvador, Georgia, Guatemala, Honduras, India, Indonesia, Kenya, Moldova, Morocco, Philippines, Ukraine, Zambia.
- Upper Middle-Income Countries: Algeria, Brazil, Bulgaria, China, Colombia, Costa Rica, Ecuador, Jordan, Malaysia, Mexico, Panama, Peru, Romania, South Africa, Tunisia, Turkey.
- High-Income Countries: Argentina, Australia, Austria, Belgium, Canada, Chile, Croatia, Czech Republic, Denmark, Estonia, Finland, France, Germany, Greece, Hong-Kong SAR/China, Hungary, Ireland, Israel, Italy, Japan, Korea (Rep.), Latvia, Lithuania, Netherlands, New Zealand, Norway, Poland, Portugal, Russian Federation, Saudi Arabia, Slovak Republic, Slovenia, Spain, Sweden, Switzerland, United Kingdom, United States, Uruguay.

(a) Directly received food innovations, by country and year

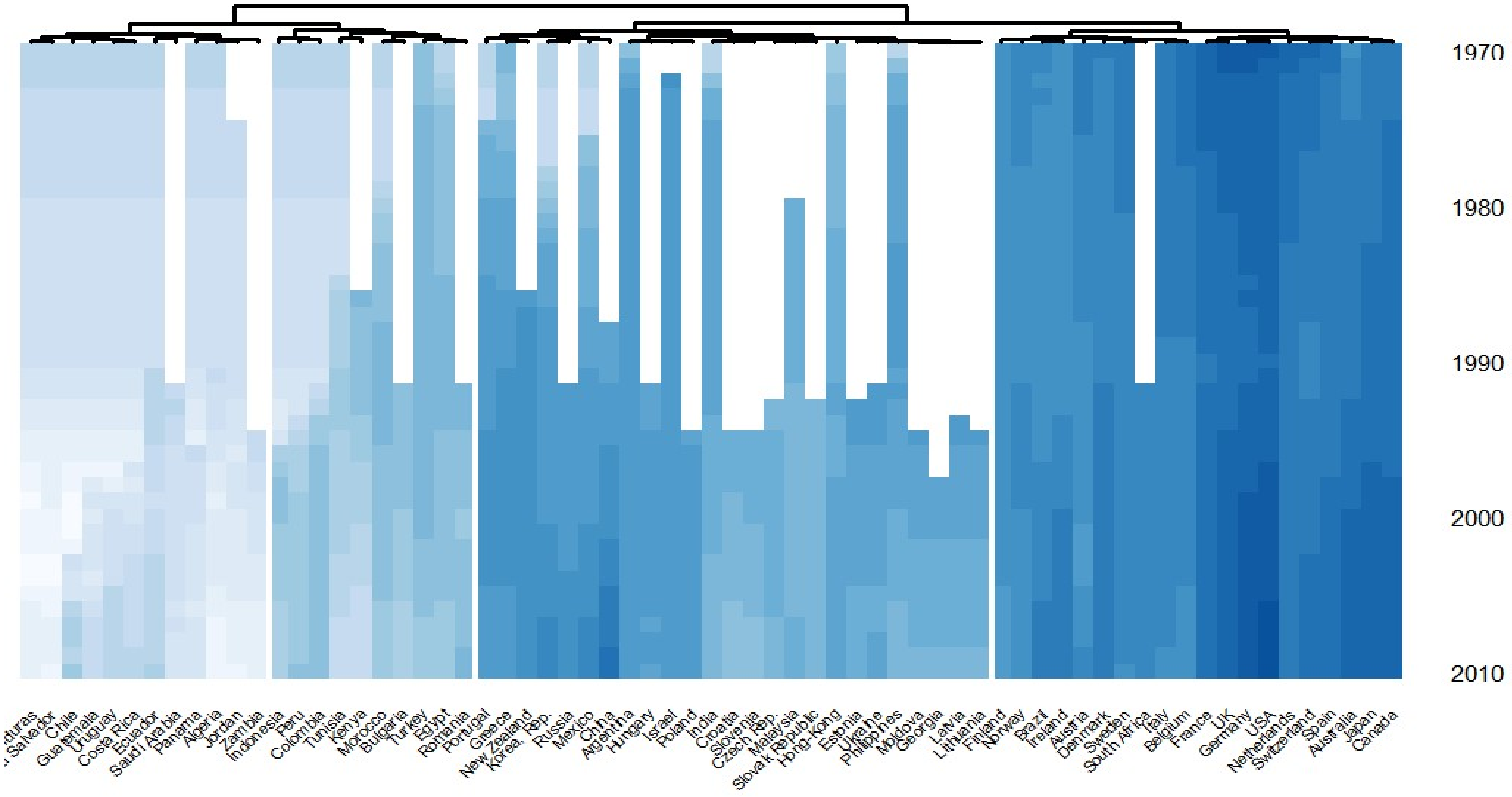

Note: The dendrogram groups countries with similar patterns in terms of food innovations into common clusters. The darker the more patent applications there are in the country. This dendogram was produced by applying the R function *heatmap*.2 to the variable $PATENTSTOCK_{ct}$ in the unbalanced sample of country-year observations.

(b) Indirectly imported food innovations, by country and year

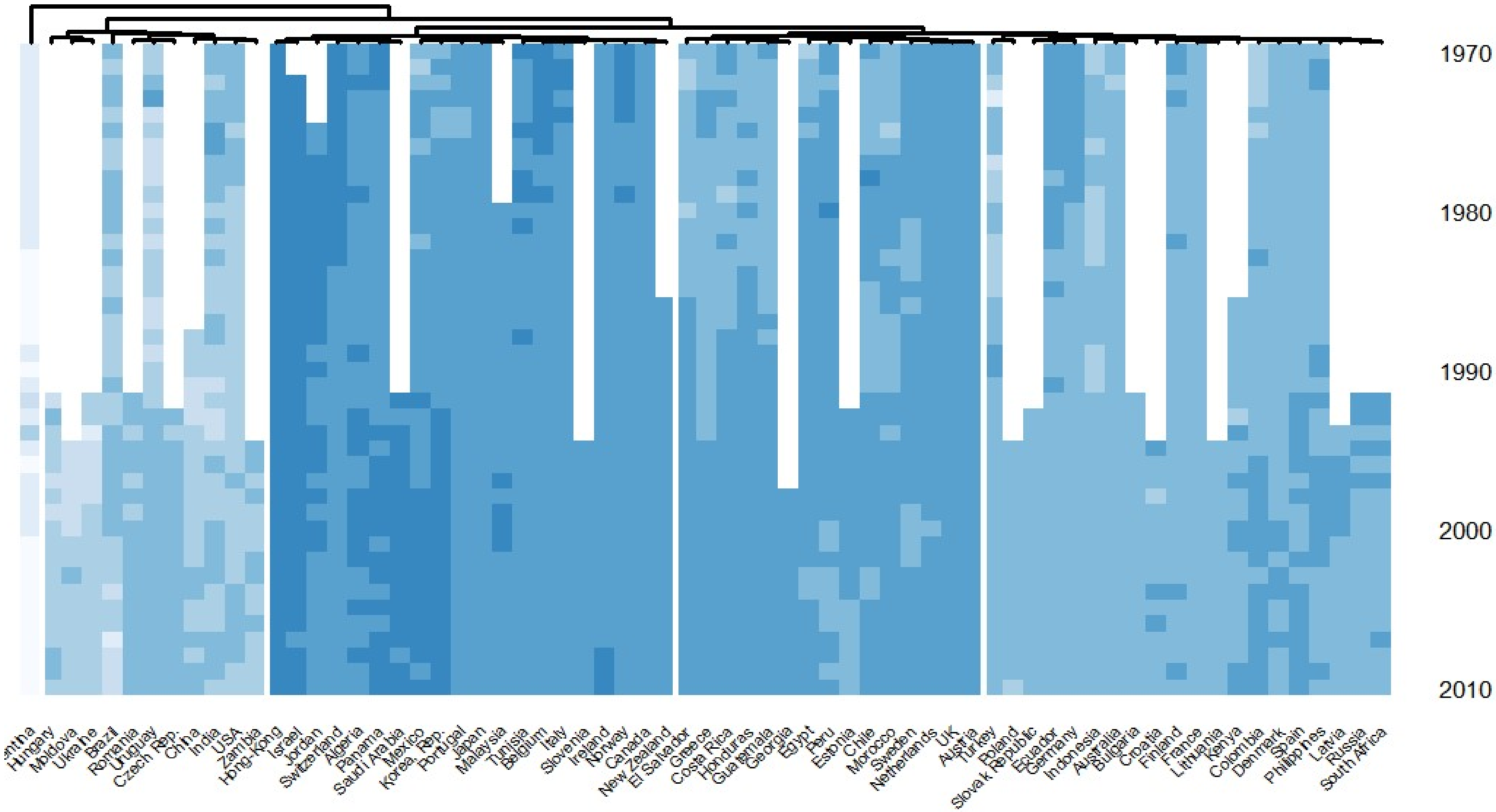

Note: this dendogram was produced by applying the R function *heatmap*.2 to the variable $PATENTTRADESTOCK_{ct} = \sum_{c' \neq c}(SHAREIMPORTPROCESS_{cc't} \times PATENTSTOCK_{c't})$ in the unbalanced sample of country-year observations.

(c) Total food innovations, by country and year

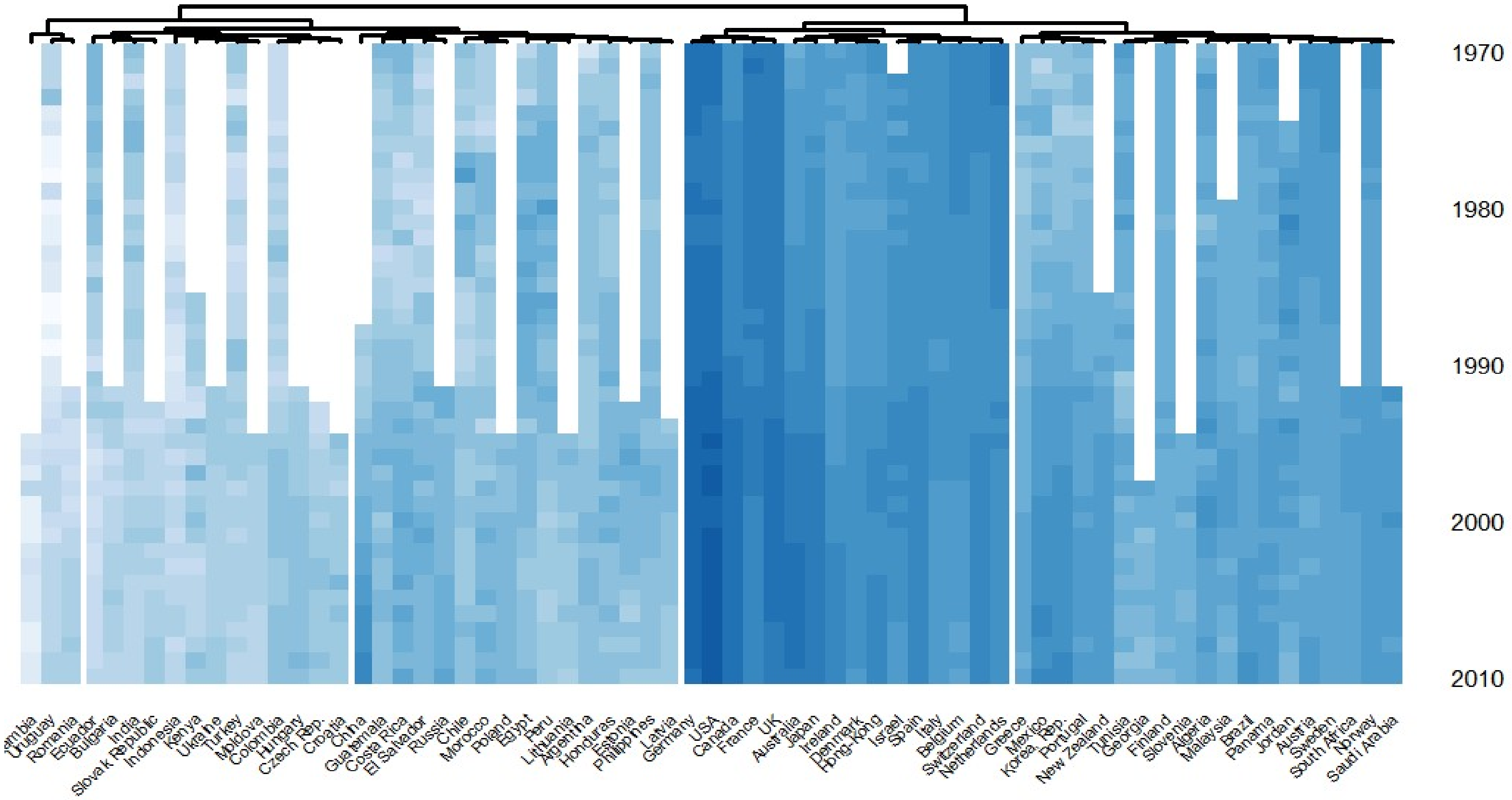

Note: this dendogram was produced by applying the R function *heatmap*.2 to the variable $INNOVSTOCK_{ct}$ in the unbalanced sample of country-year observations.

Supplementary Figure 4 – Dendrograms: Patterns of technology diffusion

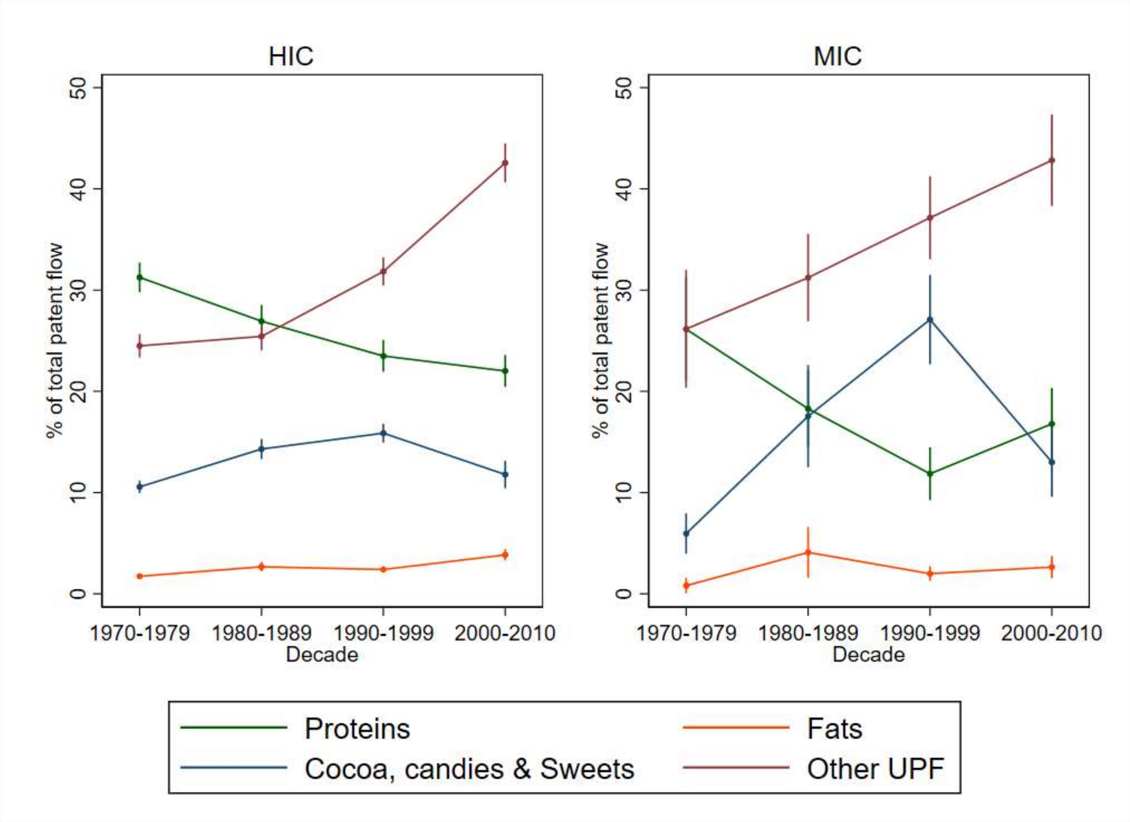

Note: This figure shows the share in the total patents of patents related to proteins, cocoa, candies and sweets, fat, and other ultra-processed food products in high-income countries (HIC) and middle-income countries (MIC) between 1970 and 2010. Average flows for each decade are reported. Proteins: IPC codes A22B, A22C, A23C, A23J; Fats: IPC code A23D; Cocoa, candies & sweets: IPC code A23G; Other Ultra-Processed Food (UPF): IPC codes A23L, A23P. 95% confidence interval plots.

Supplementary Figure 5 – Trends in food innovation categories

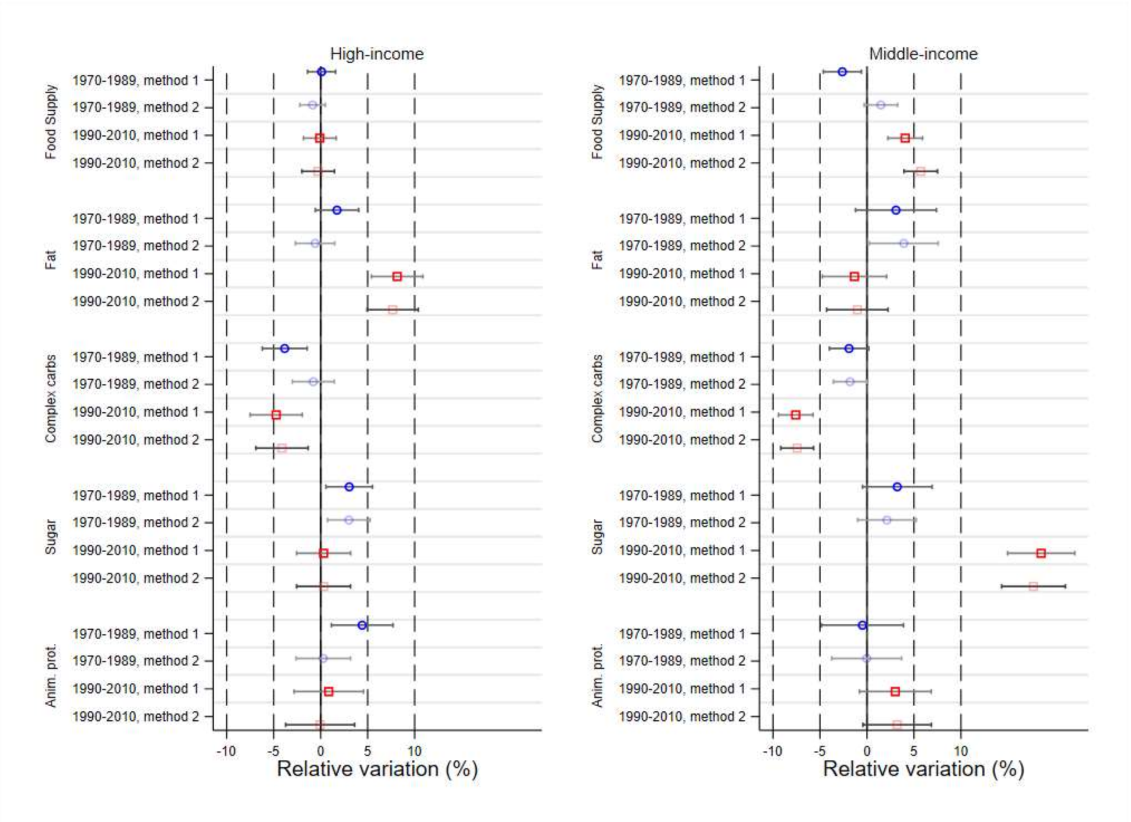

Note: method 1 accounts for Patent Cooperation Treaty (PCT) and Paris Convention memberships (as in Figure 2 in the main text); Method 2 ignores PCT and Paris Convention memberships.

Supplementary Figure 6. Simulated changes in nutritional outcomes if food innovations stock increases up to the technological frontier – Sensitivity analysis varying the calculation method.

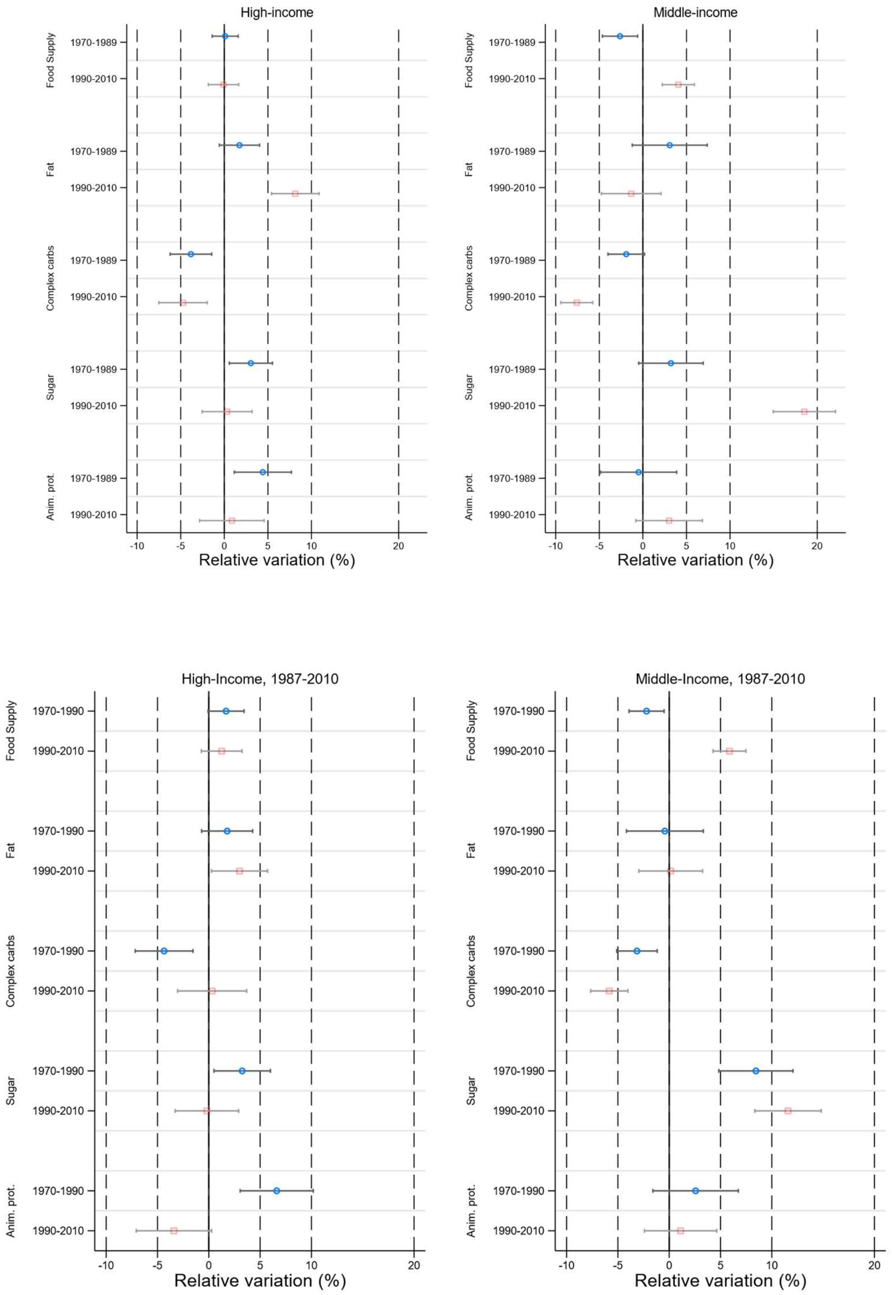

Note: The upper panel reproduces Figure 2 in the main text. It shows the effect of setting countries at the technological frontier of their income group, where countries are defined as HIC or MIC depending on their World Bank classification in 2014. The lower panel represents the same effect when countries are defined as HIC or MIC depending on their *modal* World Bank classification over the period 1987-2010. Twelve countries move from the HIC to the UMI category: Argentina, Chile, Croatia, Czech Republic, Estonia, Hungary, Latvia, Lithuania, Poland, Saudi Arabia, Slovak Republic, Uruguay. Russian federation falls in the LMI category, and eleven countries move from the UMI to LMI category: Algeria, Bulgaria, China, Colombia, Costa Rica, Ecuador, Jordan, Peru, Romania, Tunisia, Turkey.

Supplementary Figure 7. Simulated changes in nutritional outcomes if food innovations stock increases up to the technological frontier – Sensitivity analysis varying the definition of income groups.

Disdier A-C, *et al. BMJ Glob Health* 2023; 8:e012062. doi: 10.1136/bmjgh-2023-012062

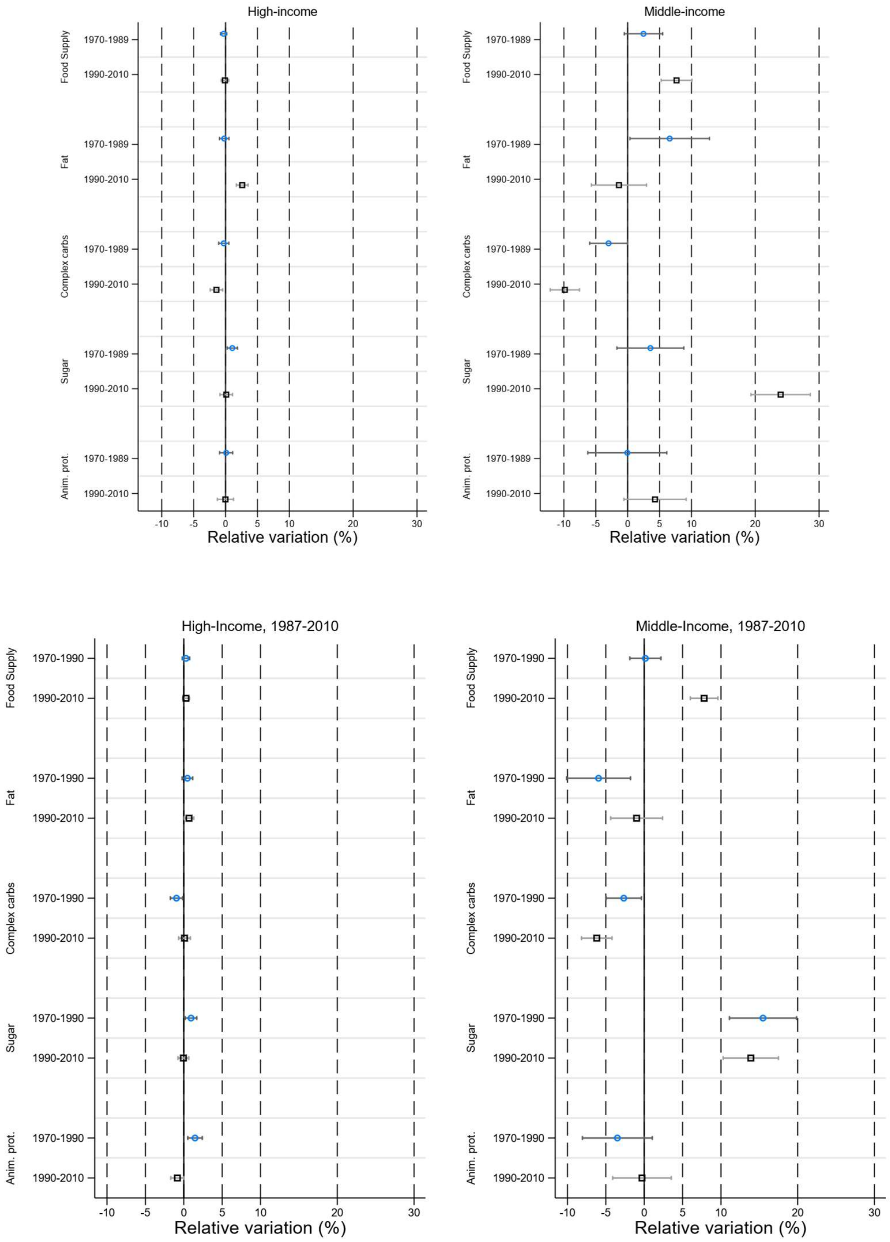

Note: The upper panel reproduces Figure 3 in the main text. It illustrates the contribution of the trade channel to the diet, when countries are defined as HIC or MIC depending on their World Bank classification in 2014. The lower panel represents the same effect when countries are defined as HIC or MIC depending on their *modal* World Bank classification over the period 1987-2010. Twelve countries move from the HIC to UMI category: Argentina, Chile, Croatia, Czech Republic, Estonia, Hungary, Latvia, Lithuania, Poland, Saudi Arabia, Slovak Republic, Uruguay. Russian federation falls in the LMI category, and eleven countries move from the UMI to the LMI category: Algeria, Bulgaria, China, Colombia, Costa Rica, Ecuador, Jordan, Peru, Romania, Tunisia, Turkey.

Supplementary Figure 8. Contribution of international trade to changes in nutritional outcomes – Sensitivity analysis varying the definition of income groups.

Supplementary Table 1. List of relevant food codes based on the International Patent Classification (IPC)

4-digit code	*Coverage*
A21B	BAKING; EQUIPMENT FOR MAKING OR PROCESSING DOUGHS; DOUGHS FOR BAKING (excludes domestic equipments and combustion apparatus)
A21C	MACHINES OR EQUIPMENT FOR MAKING OR PROCESSING DOUGHS; HANDLING BAKED ARTICLES MADE FROM DOUGH
A21D	TREATMENT, e.g. PRESERVATION, OF FLOUR OR DOUGH FOR BAKING, e.g. BY ADDITION OF MATERIALS; BAKING; BAKERY PRODUCTS; PRESERVATION THEREOF
A22B	SLAUGHTERING
A22C	PROCESSING MEAT, POULTRY, OR FISH (excludes: preserving ; obtaining protein composition for foodstuffs; meat, poultry or fish preparations ; disintegrating, e.g. chopping meat ; preparation of proteins in general)
A23B	PRESERVING, e.g. BY CANNING, MEAT, FISH, EGGS, FRUIT, VEGETABLES, EDIBLE SEEDS; CHEMICAL RIPENING OF FRUIT OR VEGETABLES; THE PRESERVED, RIPENED, OR CANNED PRODUCTS
A23C	DAIRY PRODUCTS, e.g. MILK, BUTTER, CHEESE; MILK OR CHEESE SUBSTITUTES; MAKING THEREOF (excludes: obtaining protein compositions for foodstuffs; preparation of peptides, e.g. of proteins, in general)
A23D	EDIBLE OILS OR FATS, e.g. MARGARINES, SHORTENINGS, COOKING OILS (excludes : obtaining, refining, preserving; hydrogenation)
A23F	COFFEE ; TEA ; THEIR SUBSTITUTES ; MANUFACTURE, PREPARATION, OR INFUSION THEREOF
A23G	COCOA; COCOA PRODUCTS, e.g. CHOCOLATE; SUBSTITUTES FOR COCOA OR COCOA PRODUCTS; CONFECTIONERY; CHEWING GUM; ICE-CREAM; PREPARATION THEREOF
A23J	PROTEIN COMPOSITIONS FOR FOODSTUFFS; WORKING-UP PROTEINS FOR FOODSTUFFS; PHOSPHATIDE COMPOSITIONS FOR FOODSTUFFS
A23L	FOODS, FOODSTUFFS, OR NON-ALCOHOLIC BEVERAGES, NOT COVERED BY SUBCLASSES A21D OR A23B-A23J ; THEIR PREPARATION OR TREATMENT, e.g. COOKING, MODIFICATION OF NUTRITIVE QUALITIES, PHYSICAL TREATMENT (shaping or working, not fully covered by this subclass, A23P) ; PRESERVATION OF FOODS OR FOODSTUFFS, IN GENERAL (preservation of flour or dough for baking A21D)
A23P	SHAPING OR WORKING OF FOODSTUFFS, NOT FULLY COVERED BY A SINGLE OTHER SUBCLASS
C12C	BREWING OF BEER
C12F	RECOVERY OF BY-PRODUCTS OF FERMENTED SOLUTIONS
C12G	WINE ; OTHER ALCOHOLIC BEVERAGES
C12H	PASTEURISATION, STERILISATION, PRESERVATION, PURIFICATION, CLARIFICATION, AGEING OF ALCOHOLIC BEVERAGES OR REMOVAL OF ALCOHOL THEREFROM
C12J	VINEGAR
C13B	PRODUCTION OF SUCROSE; APPARATUS SPECIALLY ADAPTED THEREFORE
C13K	GLUCOSE ; INVERT SUGAR ; LACTOSE ; MALTOSE, SYNTHESIS OF SUGARS BY HYDROLYSIS OF DI- OR POLYSACCHARIDES

Source: https://www.wipo.int/classifications/ipc/en/

Supplementary Table 2. Assignment of items to nutrient categories

Food item (FAO nomenclature)	Free fats	Vegetable fats	Animal fats	Complex carbs	Sugars	Animal proteins	Vegetable proteins
Wheat and products	0	1	0	1	0	0	1
Barley and products	0	1	0	1	0	0	1
Maize and products	0	1	0	1	0	0	1
Rye and products	0	1	0	1	0	0	1
Oats	0	1	0	1	0	0	1
Millet and products	0	1	0	1	0	0	1
Sorghum and products	0	1	0	1	0	0	1
Cereals, Other	0	1	0	1	0	0	1
Potatoes and products	0	1	0	1	0	0	1
Cassava and products	0	1	0	1	0	0	1
Sweet potatoes	0	1	0	1	0	0	1
Roots, Other	0	1	0	1	0	0	1
Yams	0	1	0	1	0	0	1
Sugar cane	1	0	0	0	1	0	1
Sugar beet	1	0	0	0	1	0	1
Sugar non-centrifugal	0	1	0	0	1	0	1
Sugar (Raw Equivalent)	0	1	0	0	1	0	1
Sweeteners, Other	0	1	0	0	1	0	1
Beans	0	1	0	1	0	0	1
Peas	0	1	0	1	0	0	1
Pulses, Other and products	0	1	0	1	0	0	1
Soyabeans	0	1	0	1	0	0	1
Groundnuts (Shelled Eq)	0	1	0	1	0	0	1
Sunflower seed	0	1	0	1	0	0	1
Rape and Mustardseed	0	1	0	1	0	0	1
Cottonseed	0	1	0	1	0	0	1
Coconuts - Incl Copra	0	1	0	1	0	0	1
Sesame seed	0	1	0	1	0	0	1
Palm kernels	0	1	0	1	0	0	1
Olives (including preserved)	0	1	0	1	0	0	1
Oilcrops, Other	0	1	0	1	0	0	1
Soyabean Oil	1	0	0	1	0	0	1
Groundnut Oil	1	0	0	1	0	0	1
Sunflowerseed Oil	1	0	0	1	0	0	1
Rape and Mustard Oil	1	0	0	1	0	0	1
Cottonseed Oil	1	0	0	1	0	0	1
Palmkernel Oil	1	0	0	1	0	0	1
Palm Oil	1	0	0	1	0	0	1
Coconut Oil	1	0	0	1	0	0	1
Sesameseed Oil	1	0	0	1	0	0	1
Olive Oil	1	0	0	1	0	0	1
Ricebran Oil	1	0	0	1	0	0	1
Maize Germ Oil	1	0	0	1	0	0	1
Oilcrops Oil, Other	1	0	0	1	0	0	1
Tomatoes and products	0	1	0	0	1	0	1
Onions	0	1	0	0	1	0	1
Vegetables, Other	0	1	0	0	1	0	1
Oranges, Mandarines	0	1	0	0	1	0	1
Lemons, Limes and products	0	1	0	0	1	0	1
Grapefruit and products	0	1	0	0	1	0	1
Citrus, Other	0	1	0	0	1	0	1
Bananas	0	1	0	0	1	0	1
Plantains	0	1	0	0	1	0	1
Apples and products	0	1	0	0	1	0	1
Pineapples and products	0	1	0	0	1	0	1
Dates	0	1	0	0	1	0	1

Grapes, products (excl. wine)	0	1	0	0	1	0	1
Fruits, Other	0	1	0	0	1	0	1
Coffee and products	0	0	0	0	0	0	1
Cocoa Beans and products	0	0	0	0	0	0	1
Tea (including mate)	0	0	0	0	0	0	1
Pepper	0	0	0	0	0	0	1
Pimento	0	0	0	0	0	0	1
Cloves	0	0	0	0	0	0	1
Spices, Other	0	0	0	0	0	0	1
Wine	0	0	0	0	1	0	1
Beer	0	0	0	0	1	0	1
Beverages, Fermented	0	0	0	0	1	0	1
Beverages, Alcoholic	0	0	0	0	1	0	1
Alcohol, Non-Food	0	0	0	0	1	0	1
Bovine Meat	0	0	1	1	0	1	0
Mutton & Goat Meat	0	0	1	1	0	1	0
Pigmeat	0	0	1	1	0	1	0
Poultry Meat	0	0	1	1	0	1	0
Meat, Other	0	0	1	1	0	1	0
Fats, Animals, Raw	1	0	0	1	0	1	0
Butter, Ghee	1	0	0	1	0	1	0
Cream	1	0	0	1	0	1	0
Honey	0	1	0	0	1	0	1
Freshwater Fish	0	0	1	1	0	1	0
Demersal Fish	0	0	1	1	0	1	0
Pelagic Fish	0	0	1	1	0	1	0
Marine Fish, Other	0	0	1	1	0	1	0
Crustaceans	0	0	1	1	0	1	0
Cephalopods	0	0	1	1	0	1	0
Molluscs, Other	0	0	1	1	0	1	0
Meat, Aquatic Mammals	0	0	1	1	0	1	0
Aquatic Animals, Others	0	0	1	1	0	1	0
Aquatic Plants	0	1	0	1	0	1	0
Fish, Body Oil	1	0	0	1	0	1	0
Fish, Liver Oil	1	0	0	1	0	1	0
Rice (Milled Equivalent)	0	1	0	1	0	0	1
Cereals - excluding beer	0	1	0	1	0	0	1
Amyl roots + (Total)	0	1	0	1	0	0	1
Sugar cultures + (Total)	1	0	0	0	1	0	1
Sugar & stimulants + (Total)	0	1	0	0	1	0	1
Legumes + (Total)	0	1	0	1	0	0	1
Hull fruits + (Total)	0	1	0	1	0	0	1
Oleaginous plant + (Total)	0	1	0	1	0	0	1
Vegetable oils + (Total)	1	0	0	1	0	0	1
Vegetables + (Totals)	0	1	0	0	1	0	1
Fruits, excluding wine + (Total)	0	1	0	0	1	0	1
Stimulants + (Total)	0	0	0	0	0	0	1
Spices + (Total)	0	0	0	0	0	0	1
Alcoholic beverages + (Total)	0	0	0	0	1	0	1
Diverse products	1	1	0	1	0	0	1
Meat products + (Total)	0	0	1	1	0	1	0
Giblets + (Total)	0	0	1	1	0	1	0
Animal fats + (Total)	1	0	0	1	0	1	0
Milk, excluding butter + (Total)	0	0	1	0	1	1	0
Eggs + (Total)	0	0	1	1	0	1	0
Fish and sea food + (Total)	0	0	1	1	0	1	0
Aquatic products, others + (Total)	0	0	1	1	0	1	0

Note: assignment as in Oberlander, L., Disdier, A. C., & Etilé, F. (2017). Globalization and national trends in nutrition and health: A grouped fixed-effects approach to intercountry heterogeneity. *Health Economics*, *26*(9), 1146-1161.

Supplementary Table 3. Associations (estimated coefficients) between food innovations received by HIC and food supply and its main components, controlling for other covariates

	Food Supply	Carbs	Complex carbs	Sugars	Fats	Vegetable fats	Free or animal fats	Proteins	Vegetable proteins	Animal proteins
	(1)	(2)	(3)	(4)	(5)	(6)	(7)	(8)	(9)	(10)
Ln total patents	-0.004	0.004	-0.005	0.018***	-0.004	0.005	-0.005	0.002	0.007	0.001
	(0.003)	(0.007)	(0.007)	(0.007)	(0.007)	(0.008)	(0.008)	(0.004)	(0.005)	(0.007)
Ln total patents x period 1990-2010	0.003	-0.038***	-0.021***	-0.016***	0.055***	-0.030***	0.069***	-0.015***	-0.017***	-0.002
	(0.003)	(0.005)	(0.006)	(0.006)	(0.006)	(0.007)	(0.007)	(0.003)	(0.004)	(0.006)
Ln GDP per capita	0.075***	-0.547***	-0.880***	0.671***	0.885***	-0.311***	1.237***	-0.148***	-0.407***	0.566***
	(0.022)	(0.046)	(0.050)	(0.047)	(0.050)	(0.056)	(0.057)	(0.025)	(0.034)	(0.049)
Ln squared GDP per capita	-0.003	0.071***	0.106***	-0.084***	-0.127***	0.062***	-0.184***	0.037***	0.051***	-0.059***
	(0.004)	(0.009)	(0.010)	(0.009)	(0.010)	(0.011)	(0.011)	(0.005)	(0.007)	(0.010)
WLFP (%)	0.001	-0.007***	-0.015***	-0.001	0.005**	-0.003	0.001	-0.001	-0.013***	0.003
	(0.001)	(0.002)	(0.002)	(0.002)	(0.002)	(0.002)	(0.003)	(0.001)	(0.002)	(0.002)
WLFP squared	-0.000	0.000***	0.000***	0.000	-0.000***	0.000**	-0.000**	0.000	0.000***	-0.000**
	(0.000)	(0.000)	(0.000)	(0.000)	(0.000)	(0.000)	(0.000)	(0.000)	(0.000)	(0.000)
Urbanization rate (%)	-0.003***	-0.004***	-0.005***	0.003***	0.006***	-0.007***	0.011***	0.003***	-0.001**	0.010***
	(0.000)	(0.001)	(0.001)	(0.001)	(0.001)	(0.001)	(0.001)	(0.000)	(0.001)	(0.001)
Social globalization	0.001***	-0.004***	-0.002*	-0.005***	0.003***	-0.003***	0.002**	0.001	-0.001*	0.000
	(0.000)	(0.001)	(0.001)	(0.001)	(0.001)	(0.001)	(0.001)	(0.000)	(0.001)	(0.001)
Economic globalization	0.000	0.000	0.001*	-0.001**	-0.001*	0.000	-0.001*	0.001***	0.001**	0.001***
	(0.000)	(0.000)	(0.000)	(0.000)	(0.000)	(0.000)	(0.001)	(0.000)	(0.000)	(0.000)
Ln share food imports	0.000	-0.011*	-0.006	-0.008	0.011	0.033***	0.011	0.002	-0.000	0.004
	(0.003)	(0.006)	(0.007)	(0.006)	(0.007)	(0.007)	(0.007)	(0.003)	(0.005)	(0.006)
PCT membership (0/1)	0.016***	-0.050***	-0.063***	0.002	0.050***	0.023**	0.055***	0.013***	-0.027***	0.046***
	(0.004)	(0.009)	(0.009)	(0.009)	(0.009)	(0.010)	(0.011)	(0.005)	(0.006)	(0.009)
Paris Convention membership (0/1)	-0.015**	-0.029*	-0.018	-0.008	0.008	0.046**	0.005	0.062***	-0.013	0.131***
	(0.008)	(0.016)	(0.018)	(0.017)	(0.018)	(0.020)	(0.020)	(0.009)	(0.012)	(0.017)
Observations	1,243	1,243	1,243	1,243	1,243	1,243	1,243	1,243	1,243	1,243
Countries	38	38	38	38	38	38	38	38	38	38

Note: *: p < 0.1; **: p < 0.05; ***: p < 0.01. PCT: Patent Cooperation Treaty. WLFP. Women labour force participation. The dependent variables in the first row are in log or logit form (see Equation 1 in Section B.2 of this Supplementary Appendix). The estimations also include country- and year fixed effects (not reported).

Supplementary Table 4. Associations (estimated coefficients) between food innovations received by MIC and food supply and its main components, controlling for other covariates

	Food Supply	Carbs	Complex carbs	Sugars	Fats	Vegetable fats	Free or animal fats	Proteins	Vegetable proteins	Animal proteins
	(1)	(2)	(3)	(4)	(5)	(6)	(7)	(8)	(9)	(10)
Ln total patents	0.006	-0.013*	-0.015**	0.011	0.020**	0.006	0.027**	-0.004	-0.002	-0.000
	(0.004)	(0.007)	(0.008)	(0.009)	(0.010)	(0.008)	(0.012)	(0.003)	(0.004)	(0.008)
Ln total patents x period 1990-2010	0.021***	0.021**	-0.055***	0.091***	-0.027**	0.033***	-0.033**	0.001	-0.010**	0.016*
	(0.004)	(0.008)	(0.009)	(0.010)	(0.011)	(0.010)	(0.014)	(0.004)	(0.005)	(0.010)
Ln GDP per capita	0.089***	-0.053**	-0.144***	0.240***	0.049	0.015	0.073*	0.050***	-0.008	0.353***
	(0.013)	(0.025)	(0.026)	(0.029)	(0.033)	(0.029)	(0.042)	(0.011)	(0.014)	(0.028)
Ln squared GDP per capita	-0.020***	0.045***	0.002	0.021	-0.069***	-0.020	-0.093***	0.013**	0.001	-0.027**
	(0.006)	(0.012)	(0.012)	(0.014)	(0.016)	(0.014)	(0.020)	(0.005)	(0.007)	(0.013)
WLFP (%)	0.001*	-0.002	-0.002	0.003*	0.002	-0.012***	0.003	0.001	-0.004***	0.012***
	(0.001)	(0.002)	(0.002)	(0.002)	(0.002)	(0.002)	(0.003)	(0.001)	(0.001)	(0.002)
WLFP squared	-0.000	0.000	0.000	-0.000***	-0.000	0.000***	-0.000	0.000	0.000***	-0.000***
	(0.000)	(0.000)	(0.000)	(0.000)	(0.000)	(0.000)	(0.000)	(0.000)	(0.000)	(0.000)
Urbanization rate (%)	0.001	-0.006***	-0.004***	0.001	0.007***	-0.004***	0.012***	0.002***	0.001	0.007***
	(0.001)	(0.001)	(0.001)	(0.001)	(0.001)	(0.001)	(0.002)	(0.001)	(0.001)	(0.001)
Social globalization	-0.001	-0.004***	-0.003***	0.000	0.004***	-0.003***	0.005***	0.002***	-0.001	0.004***
	(0.001)	(0.001)	(0.001)	(0.001)	(0.001)	(0.001)	(0.002)	(0.000)	(0.001)	(0.001)
Economic globalization	-0.000	-0.001*	-0.001	-0.000	0.002**	-0.001**	0.002***	-0.001***	-0.001***	-0.002***
	(0.000)	(0.000)	(0.000)	(0.001)	(0.001)	(0.001)	(0.001)	(0.000)	(0.000)	(0.001)
Ln share food imports	-0.002	-0.000	-0.013*	0.020***	0.009	0.005	0.010	-0.012***	-0.003	-0.026***
	(0.003)	(0.007)	(0.007)	(0.008)	(0.009)	(0.008)	(0.011)	(0.003)	(0.004)	(0.007)
PCT membership (0/1)	-0.031***	0.008	-0.010	0.026**	-0.008	-0.054***	-0.004	-0.002	-0.010	-0.005
	(0.006)	(0.011)	(0.012)	(0.013)	(0.015)	(0.013)	(0.019)	(0.005)	(0.006)	(0.013)
Paris Convention membership (0/1)	-0.022***	0.002	0.015	-0.023	-0.004	-0.099***	0.034	0.010*	-0.001	-0.000
	(0.007)	(0.013)	(0.013)	(0.015)	(0.017)	(0.015)	(0.021)	(0.006)	(0.007)	(0.014)
Observations	945	945	945	945	945	945	945	945	945	945
Countries	29	29	29	29	29	29	29	29	29	29

Note: *: p < 0.1; **: p < 0.05; ***: p < 0.01. PCT: Patent Cooperation Treaty. WLFP. Women labour force participation. The dependent variables in the first row are in log or logit form (see Equation 1 in Section B.2 of this Supplementary Appendix). The estimations also include country- and year x income group fixed effects (not reported).

Supplementary Table 5. Associations (elasticities) between food innovations received by HIC and food supply and its main components, under different specifications

Specification	Baseline	S1	S2	S3	S4	S5
Food supply, 1970-1989	-0.004	-0.004	-0.007**	0.017**	-0.002	-0.001
	(0.003)	(0.003)	(0.003)	(0.009)	(0.003)	(0.002)
Food supply, 1990-2010	-0.001	0.000	-0.003	0.019**	-0.000	-0.002
	(0.004)	(0.004)	(0.004)	(0.009)	(0.004)	(0.002)
Carbohydrates, 1970-1989	0.002	0.003	-0.002	0.013	0.000	-0.003
	(0.003)	(0.003)	(0.003)	(0.012)	(0.003)	(0.002)
Carbohydrates, 1990-2010	-0.016***	-0.017***	-0.019***	0.013	-0.017***	-0.007***
	(0.004)	(0.004)	(0.004)	(0.012)	(0.004)	(0.002)
Complex carbs. ,1970-1989	-0.004	-0.002	-0.006	0.010	-0.009*	-0.006*
	(0.005)	(0.005)	(0.005)	(0.020)	(0.005)	(0.003)
Complex carbs., 1990-2010	-0.018***	-0.021***	-0.017***	0.012	-0.027***	-0.009***
	(0.006)	(0.007)	(0.006)	(0.020)	(0.007)	(0.003)
Sugars, 1970-1989	0.013***	0.014***	0.013***	0.000	0.016***	0.002
	(0.005)	(0.005)	(0.005)	(0.019)	(0.004)	(0.003)
Sugars, 1990-2010	0.001	0.002	-0.003	-0.002	0.009	0.002
	(0.006)	(0.006)	(0.006)	(0.019)	(0.006)	(0.003)
Fats, 1970-1989	-0.003	-0.004	0.005	-0.028	-0.001	0.001
	(0.005)	(0.005)	(0.005)	(0.020)	(0.005)	(0.003)
Fats, 1990-2010	0.033***	0.035***	0.040***	-0.028	0.033***	0.013***
	(0.006)	(0.006)	(0.006)	(0.020)	(0.006)	(0.003)
Vegetable fats, 1970-1989	0.005	0.003	-0.008	0.075	-0.003	-0.007
	(0.008)	(0.008)	(0.008)	(0.048)	(0.008)	(0.005)
Vegetable fats, 1990-2010	-0.025**	-0.025**	-0.037***	0.079*	-0.035***	-0.018***
	(0.010)	(0.010)	(0.010)	(0.046)	(0.010)	(0.005)
Free or animal fats, 1970-1989	-0.004	-0.005	0.010*	-0.043	-0.002	0.001
	(0.006)	(0.006)	(0.006)	(0.027)	(0.006)	(0.004)
Free or animal fats, 1990-2010	0.044***	0.046***	0.057***	-0.043	0.045***	0.021***
	(0.007)	(0.007)	(0.007)	(0.027)	(0.007)	(0.004)
Proteins, 1970-1989	0.002	0.000	0.006*	0.006	0.007**	0.007***
	(0.003)	(0.003)	(0.003)	(0.010)	(0.003)	(0.002)
Proteins, 1990-2010	-0.011***	-0.012***	-0.007*	0.007	-0.007	-0.001
	(0.004)	(0.004)	(0.004)	(0.011)	(0.004)	(0.002)
Vegetable proteins, 1970-1989	0.006	0.007	0.007	0.023	0.004	-0.004
	(0.005)	(0.005)	(0.005)	(0.023)	(0.005)	(0.003)
Vegetable proteins, 1990-2010	-0.009	-0.010*	-0.004	0.026	-0.013**	-0.004
	(0.006)	(0.006)	(0.006)	(0.022)	(0.006)	(0.003)
Animal proteins, 1970-1989	0.001	-0.003	0.013**	-0.021	0.010	0.015***
	(0.007)	(0.007)	(0.007)	(0.030)	(0.006)	(0.004)
Animal proteins, 1990-2010	-0.000	-0.002	0.008	-0.022	0.008	0.009**
	(0.008)	(0.009)	(0.008)	(0.030)	(0.008)	(0.004)
Base controls	Yes	Yes	Yes	Yes	Yes	Yes
Additional controls	Yes	Yes	No	No	Yes	Yes
PCT/Paris Convention	Yes	No	Yes	Yes	Yes	Yes
Country fixed effects	Yes	Yes	Yes	No	Yes	Yes
Year fixed effects	Yes	Yes	Yes	No	Yes	Yes

Disdier A-C, *et al. BMJ Glob Health* 2023; 8:e012062. doi: 10.1136/bmjgh-2023-012062

Balanced panel	No	No	No	No	Yes	No
Include trade imported patents	Yes	Yes	Yes	Yes	Yes	No
Observations	1,243	1,243	1,243	1,243	1000	1,243
Countries	38	38	38	38	25	38

Note: *: p < 0.1; **: p < 0.05; ***: p < 0.01. Base controls: Ln GDP per capita, ln squared GDP per capita, ln share food imports; Additional controls: Economic globalization, social globalization, women labour force participation (%), women labour force participation squared, urbanization rate (%). PCT/Paris Convention: dummy variables for Patent Cooperation Treaty (PCT) and Paris Convention memberships. Elasticities are computed using formula provided in Section B.3 of this Supplementary Appendix.

Baseline: estimates from Table 2

S1: do not adjust for memberships of PCT or Paris convention

S2: do not adjust for additional control variables

S3: do adjust neither for country and year fixed effects, nor for additional control variables

S4: baseline specification estimated in the balanced subsample of countries

S5: replace our measure of innovation (INNOVSTOCK) by the stock of patent applications of the country (PATENTSTOCK) – patents embedded in food imports are ignored.

Supplementary Table 6. Associations (elasticities) between food innovations received by MIC and food supply and its main components, under different specifications

Specification	Baseline	S1	S2	S3	S4	S5
Food supply, 1970-1989	0.006	0.003	0.007*	0.021	0.008*	-0.000
	(0.004)	(0.004)	(0.004)	(0.021)	(0.004)	(0.002)
Food supply, 1990-2010	0.027***	0.028***	0.030***	0.020	0.030***	-0.001
	(0.004)	(0.004)	(0.004)	(0.023)	(0.005)	(0.002)
Carbohydrates, 1970-1989	-0.004*	-0.004*	-0.007***	-0.008	-0.001	0.005***
	(0.002)	(0.002)	(0.002)	(0.010)	(0.002)	(0.001)
Carbohydrates, 1990-2010	0.003	0.003	0.002	-0.008	0.007**	0.003**
	(0.003)	(0.003)	(0.003)	(0.012)	(0.003)	(0.001)
Complex carbs. ,1970-1989	-0.008**	-0.008**	-0.011***	-0.016	-0.005	0.010***
	(0.004)	(0.004)	(0.004)	(0.025)	(0.004)	(0.002)
Complex carbs., 1990-2010	-0.037***	-0.037***	-0.039***	-0.018	-0.038***	0.001
	(0.004)	(0.004)	(0.004)	(0.027)	(0.005)	(0.002)
Sugars, 1970-1989	0.009	0.010	0.009	0.029	0.011	-0.001
	(0.007)	(0.007)	(0.007)	(0.055)	(0.008)	(0.004)
Sugars, 1990-2010	0.082***	0.082***	0.087***	0.034	0.087***	0.010***
	(0.008)	(0.008)	(0.007)	(0.059)	(0.009)	(0.004)
Fats, 1970-1989	0.016**	0.016**	0.024***	0.028	0.008	-0.019***
	(0.008)	(0.008)	(0.008)	(0.026)	(0.008)	(0.004)
Fats, 1990-2010	-0.005	-0.005	-0.004	0.024	-0.014	-0.009**
	(0.008)	(0.008)	(0.008)	(0.027)	(0.010)	(0.004)
Vegetable fats, 1970-1989	0.005	-0.001	-0.001	-0.079	-0.009	0.006
	(0.008)	(0.008)	(0.008)	(0.065)	(0.009)	(0.004)
Vegetable fats, 1990-2010	0.037***	0.038***	0.022**	-0.074	0.035***	0.020***
	(0.009)	(0.009)	(0.009)	(0.066)	(0.011)	(0.004)
Free or animal fats, 1970-1989	0.023**	0.023**	0.035***	0.073*	0.014	-0.024***
	(0.010)	(0.010)	(0.011)	(0.043)	(0.012)	(0.006)
Free or animal fats, 1990-2010	-0.005	-0.005	-0.003	0.066	-0.011	-0.015***
	(0.011)	(0.011)	(0.011)	(0.044)	(0.013)	(0.005)
Proteins, 1970-1989	-0.004	-0.003	0.001	0.016	-0.004	0.001
	(0.003)	(0.003)	(0.003)	(0.020)	(0.003)	(0.002)
Proteins, 1990-2010	-0.002	-0.002	0.000	0.022	-0.008**	-0.000
	(0.003)	(0.003)	(0.003)	(0.021)	(0.004)	(0.002)
Vegetable proteins, 1970-1989	-0.002	-0.003	-0.002	0.002	0.002	0.008***
	(0.004)	(0.004)	(0.004)	(0.033)	(0.004)	(0.002)
Vegetable proteins, 1990-2010	-0.011***	-0.011***	-0.015***	0.005	-0.011**	0.000
	(0.004)	(0.004)	(0.004)	(0.034)	(0.005)	(0.002)
Animal proteins, 1970-1989	-0.000	-0.001	0.015*	0.064	-0.009	0.002
	(0.008)	(0.008)	(0.008)	(0.047)	(0.009)	(0.004)
Animal proteins, 1990-2010	0.016*	0.016*	0.035***	0.074	0.000	0.002
	(0.009)	(0.009)	(0.009)	(0.048)	(0.010)	(0.004)
Base controls	Yes	Yes	Yes	Yes	Yes	Yes
Additional controls	Yes	Yes	No	No	Yes	Yes
PCT/Paris convention	Yes	No	Yes	Yes	Yes	Yes
Country fixed effects	Yes	Yes	Yes	No	Yes	Yes
Year x income group fixed effects	Yes	Yes	Yes	No	Yes	Yes

Balanced panel	No	No	No	No	Yes	No
Include trade imported patents	Yes	Yes	Yes	Yes	Yes	No
Observations	945	945	945	945	720	945
Countries	29	29	29	29	18	29

Note: *: p < 0.1; **: p < 0.05; ***: p < 0.01. Base controls: Ln GDP per capita, ln squared GDP per capita, ln share food imports; Additional controls: Economic globalization, social globalization, women labour force participation (%), women labour force participation squared, urbanization rate (%). PCT/Paris Convention: dummy variables for Patent Cooperation Treaty (PCT) and Paris Convention memberships. Elasticities are computed using formula provided in Section B.3 of this Supplementary Appendix.
Baseline: estimates from Table 2
S1: do not adjust for memberships of PCT or Paris convention
S2: do not adjust for additional control variables
S3: do adjust neither for country and year fixed effects, nor for additional control variables
S4: baseline specification estimated in the balanced subsample of countries
S5: replace our measure of innovation (INNOVSTOCK) by the stock of patent applications of the country (PATENTSTOCK) – patents embedded in food imports are ignored.
